# Efficacy of a Self-Help Web-Based Recovery Training in Improving Sleep in Workers: Randomized Controlled Trial in the General Working Population

**DOI:** 10.2196/13346

**Published:** 2020-01-07

**Authors:** Doerte Behrendt, David Daniel Ebert, Kai Spiegelhalder, Dirk Lehr

**Affiliations:** 1 Department of Health Psychology and Applied Biological Psychology Institute of Psychology Leuphana University of Lueneburg Lueneburg Germany; 2 Faculty of Behavioural and Movement Sciences, Clinical, Neuro- & Developmental Psychology Free University Amsterdam Amsterdam Germany; 3 Department of Psychiatry and Psychotherapy Medical Center - University of Freiburg, Faculty of Medicine University of Freiburg Freiburg Germany

**Keywords:** occupational health, e-mental-health, insomnia, Web-based, cognitive behavioral therapy, mediators

## Abstract

**Background:**

Sleep complaints are among the most prevalent health concerns, especially among workers, which may lead to adverse effects on health and work. Internet-delivered cognitive behavioral therapy for insomnia (iCBT-I) offers the opportunity to deliver effective solutions on a large scale. The efficacy of iCBT-I for clinical samples has been demonstrated in recent meta-analyses, and there is evidence that iCBT-I is effective in the working population with severe sleep complaints. However, to date, there is limited evidence from randomized controlled trials that iCBT-I could also be an effective tool for universal prevention among the general working population regardless of symptom severity. Although increasing evidence suggests that negatively toned cognitive activity may be a key factor for the development and maintenance of insomnia, little is known about how iCBT-I improves sleep by reducing presleep cognitive activity.

**Objective:**

This study aimed to examine the efficacy of a self-help internet-delivered recovery training, based on principles of iCBT-I tailored to the work-life domain, among the general working population. General and work-related cognitive activities were investigated as potential mediators of the intervention’s effect.

**Methods:**

A sample of 177 workers were randomized to receive either the iCBT-I (n=88) or controls (n=89). The intervention is a Web-based training consisting of six 1-week modules. As the training was self-help, participants received nothing but technical support via email. Web-based self-report assessments were scheduled at baseline, at 8 weeks, and at 6 months following randomization. The primary outcome was insomnia severity. Secondary outcomes included measures of mental health and work-related health and cognitive activity. In an exploratory analysis, general and work-related cognitive activities, measured as worry and work-related rumination, were investigated as mediators.

**Results:**

Analysis of the linear mixed effects model showed that, relative to controls, participants who received iCBT-I reported significantly lower insomnia severity scores at postintervention (between-group mean difference −4.36; 95% CI −5.59 to − 3.03; Cohen *d*=0.97) and at 6-month follow-up (between-group difference: −3.64; 95% CI −4.89 to −2.39; Cohen *d*=0.86). The overall test of group-by-time interaction was significant (*P*<.001). Significant differences, with small-to-large effect sizes, were also detected for cognitive activity and for mental and work-related health, but not for absenteeism. Mediation analysis demonstrated that work-related rumination (indirect effect: a_1_b_1_=−0.80; SE=0.34; 95% boot CI −1.59 to −0.25) and worry (indirect effect: a_2_b_2_=−0.37; SE=0.19; 95% boot CI −0.85 to −0.09) mediate the intervention’s effect on sleep.

**Conclusions:**

A self-help Web-based recovery training, grounded in the principles of iCBT-I, can be effective in the general working population, both short and long term. Work-related rumination may be a particularly crucial mediator of the intervention’s effect, suggesting that tailoring interventions to the workplace, including components to reduce the work-related cognitive activity, might be important when designing recovery interventions for workers.

**Trial Registration:**

German Clinical Trials Register DRKS00007142; https://www.drks.de/DRKS00007142

## Introduction

### Background

Impaired sleep is a common complaint and among the most prevalent health concerns. In Western industrialized countries, approximately 30% to 35% of the general adult population report insomnia symptoms, and approximately 10% meet the criteria of insomnia as a disorder [1]. A similar situation exists in the German working population, among whom 1 study documented a 4-week prevalence of 9.4% for insomnia disorder [2]. In the same study, an additional 35.1% of the working population reported subclinical symptoms of insomnia [2]. The burden of impaired sleep is 2-fold. First, insomnia has a high impact on quality of life and daytime functioning, which includes increasing absenteeism [3]. These effects place workers at higher risk for workplace injuries and may adversely affect their work performance [4]. Second, insomnia is a risk factor for impaired health, including cardiovascular disease [4,5], metabolic syndrome [6], and a variety of mental disorders [7], especially depression [8]. In addition, subclinical insomnia that remains untreated or for which treatment is delayed places individuals at risk for developing clinically significant insomnia. This is important because untreated insomnia is associated with negative long-term health outcomes [9-11].

Published guidelines recommend insomnia-specific cognitive behavioral therapy (CBT-I) as first-line treatment [12], as it has been shown to produce large, sustainable effects [13]. However, CBT-I is not widely available, primarily offered in specialized research settings. Thus, disseminating CBT-I is a major public health challenge. Internet-delivered interventions have been suggested as a potential solution, as they are accessible to a greater number and a broader range of people [14]. Recent meta-analyses involving clinical samples have demonstrated that internet-delivered CBT-I (iCBT-I) is an effective alternative to face-to-face CBT-I [15-18]. Moreover, iCBT-I has been shown to improve not only insomnia but also other mental health outcomes, including symptoms of depression [19]. In addition, iCBT-I appears to be cost-effective from an employer’s perspective [20]. Especially when delivered in a self-help format without guidance, internet-based interventions have the potential to provide easy and affordable access to evidence-based interventions to a large population [21].

This said, most previous studies on iCBT-I targeted clinical samples or were conducted in an indicated prevention setting, not having the focus on the working population in general [15-18]. Consequently, results from previously published studies on iCBT-I might not be generalizable to the general working population, in which workers with severe sleep complaints and workers with lower or no sleep complaints are included.

### Prior Interventional Studies in the Working Population

Previous studies in the working population give first indications for the efficacy of face-to-face CBT-I for workers [22,23]. Moreover, recently conducted studies evaluating the efficacy of iCBT-I provided evidence that iCBT-I is also effective in improving sleep in the working population in the context of indicated prevention with moderate-to-large effects in the short term [24,25] and prevention with large effects in the long term [26] For specific occupational groups, namely teachers, large effects were found, both short and long-term [20,27,28]. Limitations of the prior studies are that they only included workers with elevated insomnia symptoms, thereby excluding a substantial portion of the working population with less severe insomnia symptoms. To the best of our knowledge, to date, only 2 other randomized controlled trials have evaluated the efficacy of self-help iCBT-I in a general working population. As both the trials include all interested workers regardless of insomnia symptoms, with moderate-to-large effects on sleep in the short term [29,30], they mimic a universal prevention approach.

Hence, there is insufficient evidence on whether iCBT-I might also be effective long term for the general working population without inclusion criteria on insomnia severity. This knowledge is important to clarify the question of whether iCBT-I should be part of universal prevention.

Besides assessing the efficacy of iCBT-I in the general working population, it is also of importance to understand the mechanisms underlying iCBT-I for workers. Considering risk factors for impaired sleep in workers, there is evidence that certain psychosocial work characteristics (eg, high job demands, low job control, and low perceived support) adversely impact sleep [31]. Increasing evidence indicates that negatively toned presleep cognitive activity may be an important mechanism for the relationship between work stressors and sleeping problems [32-36]. One path through which work stressors might impact sleep could be via work-related cognitive activity before sleep (eg, when individuals ruminate about previous problems at work). For example, Berset et al [33] found that work-related rumination is a mediator of work stressors’ effects on self-reported sleep quality. This can be explained by cognitive models of insomnia, which highlight the importance of increased negative cognitive activity in the development and maintenance of insomnia [32,37].

Another form of presleep cognitive activity is general worry about events that might occur in the future that are characterized by potential negative outcomes (eg, worrying about the anticipated consequences of sleep loss, similar to impaired performance the next day) [38]. Previous studies have shown that general worry before sleep plays a mediating role linking stress and impaired sleep [39,40].

Although general cognitive activity and work-related cognitive activity may involve common cognitive processes [41], the content of the 2 forms of cognitive activity is different. Consequently, it might be useful to apply different strategies, thereby targeting each process, when treating insomnia [42].

One finding from a previous study of this present training was that general cognitive activity pertaining to worry mediates the intervention’s effect on sleep [28]. However, evidence is missing on whether sleep improvement might also be mediated by reducing work-related cognitive activity [43,44]. As such, it also remains unclear whether adding training elements to reduce work-related cognitive activity and tailoring the intervention to the work-life domain is important and might incrementally contribute to designing interventions to improve sleep among workers.

### Aim of the Study

The primary aim of this study was to investigate the efficacy of a self-help version of iCBT-I, which is accessible to all workers who are interested in participating in a training to improve their sleep, employing both short- and long-term assessments of intervention effects.

As a secondary aim, we examined whether, in addition to the role of general cognitive activity, work-related cognitive activity is a putative mediator of the intervention’s efficacy in workers.

## Methods

### Study Design and Time Frame

**Figure 1:**
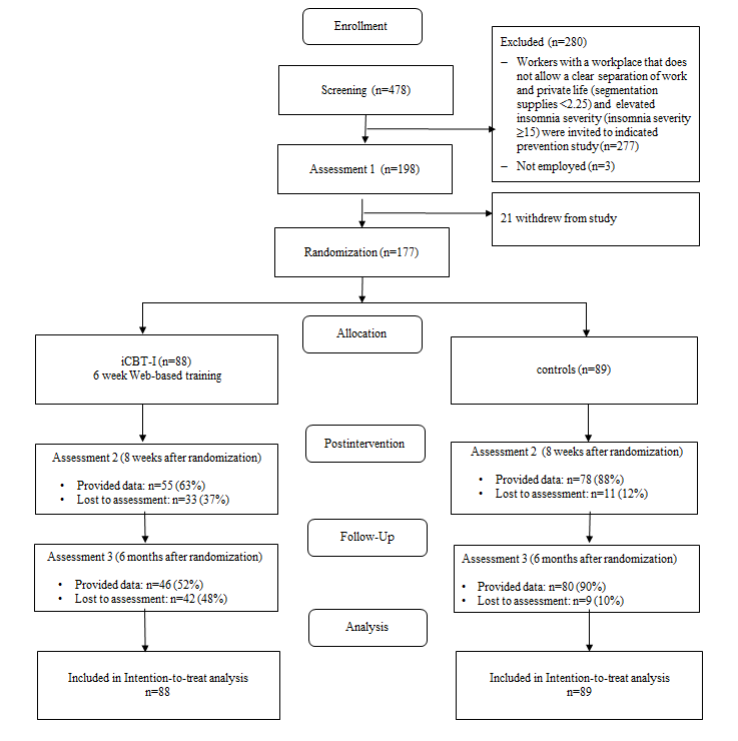
Flow of study participants. iCBT-I: internet-delivered cognitive behavioral therapy for insomnia.

In this randomized trial, subjects were randomly assigned to either receive access to an iCBT-I or received the iCBT-I 6 months later and had in the meantime full access to routine occupational health care. Primary and secondary outcomes were self-assessed Web-based at baseline (T1), postintervention (8 weeks postrandomization, T2), and at 6-month follow-up (4 months postintervention, T3; see Figure 1 for details). The Ethics Committee of Leuphana University of Lueneburg (Lehr201411_Schlaftraining) approved this study that was registered as DRKS00007142 in the German Clinical Trials Register (DRKS).

### Sample Size Calculation

Previous studies on this training have yielded intervention effects for insomnia severity ranging from *d*=1.37 to *d*=1.45 in teachers with elevated insomnia symptoms. However, studies following a universal health prevention approach without symptom severity as the inclusion criterion are usually associated with smaller effect sizes [45,46]. Thus, our sample size calculation to test the efficacy of the iCBT-I assumed an effect size of *d*=0.4 for the primary outcome (insomnia severity) at postintervention because of our inclusion of all interested workers regardless of symptom severity. We, therefore, estimated needing 200 participants to achieve 80% power (beta=.80) and 95% CI (alpha=.05) in a 2-tailed analysis.

### Inclusion and Exclusion Criteria

All workers who were aged at least 18 years, who were interested in improving their sleep through an iCBT-I, and who had access to the internet were invited to participate in the study. However, workers who expressed suicidal ideations—indicated by their response to item 9 (>1) from the Beck Depression Inventory-II [47]—were excluded. Individuals taking sleep medication were not excluded from the study but were asked to maintain a constant dose and not change their sleep medication for the full course of the study.

### Procedures

Recruitment took place via both the media (national telecast and articles) and an email distribution list from the occupational health program provided by one of the largest health insurance companies in Germany. Workers who (1) are motivated to promote their sleep, (2) want to mentally detach from problems at work, and (3) want to actively recover were addressed. Participants for 2 independent studies were recruited at the same time via the same recruiting channels. The same screening process for these 2 independent trials with different evaluation focus was used. In this trial, we evaluated the efficacy of the training in a general working population sample, where all interested workers could participate regardless of insomnia severity or any other sleep or workplace-specific characteristics.

For the other trial, workers with a workplace that is characterized by blurred boundaries between work and nonwork life (segmentation supplies <2.25) [48] and that showed a score of ≥15 of insomnia severity [49] were recruited (DRKS: DRKS00006223).

Interested workers registered for the study at the landing page of the training and had to provide an email address and a first and last name that could be pseudonyms if desired. Once registered, an individual profile on the Web-based training platform was created. Registered workers received an email with detailed information about the study and a link to the online screening questionnaire on the training platform. All individuals who (1) were employed, (2) had access to the internet, and (2) expressed no suicidal ideations were asked to complete the online baseline assessment and sign the informed consent form, after which they were randomized to 1 of the 2 intervention arms, using a computer-generated randomization list with a ratio of 1:1 and a block size of 2 [50]. The randomization list was generated, and randomization was performed blinded by 2 researchers in our department who were not otherwise involved in this study. Blinding to group allocation was not feasible. The participants were informed about the randomization outcome via email. Participants who were allocated to the training group had immediate access to the intervention. Participants in the control group received access to the training after their 6-month follow-up assessment; in the meantime, they had full access to the usual care offered by routine health care services throughout the trial.

### Intervention

In 2 previous studies, a version of the present training that was tailored to teachers was evaluated in school teachers [27,28]. The guided version of the training has been demonstrated to be effective for teachers in both the short term and long term [27]. Meanwhile, evidence supporting efficacy of the self-help version for teachers is restricted to its short-term effects only [28]. For this study, the teacher-specific version of the intervention was revised and adapted for the general working population. The intervention was developed for workers who have recurring problems initiating and maintaining sleep, who tend to ruminate about their work, and who have problems detaching from work. This present training is an internet-delivered training based on CBT-I that was specifically tailored to the needs of those workers, including elements to reduce work-related cognitive activity in the evening, in addition to well-established CBT-I methods, such as techniques to reduce general worry about anticipated consequences of sleep loss [51]. This training can be implemented in either a guided or unguided self-help format.

The training consists of six 1-week modules, each lasting 45 to 60 min. Participants could process the training the way they wanted and could access modules at any time. They were told an ideal completion time of 1 module per week. Self-help iCBT-I is beneficial, in particular, to participants with different levels of sleep complaints, as users can focus on those exercises that seem most helpful for them and can shorten others.

The modules focus on the following subjects: In module 1, subjects are provided psychoeducation on healthy sleep, plan which sleep hygiene rules they are going to follow over the subsequent week, and are introduced to the concept of a sleep diary. Module 2 focuses on sleep restriction and stimulus control. Participants are asked to plan the first step in sleep restriction and reschedule their sleep for the following week accordingly. To support this, each participant could use a Web-based sleep diary that was available on the training platform.

In module 3, participants review their progress on sleep restriction and sleep hygiene and then schedule their sleep for the next week according to their sleep restriction plan. Besides, boundary tactics to support mental detachment from work are targeted; and a gratitude diary is introduced to prevent ruminative thoughts before sleeping. During module 4, participants receive information on work-related rumination and worry, including their impact on sleep, and techniques (eg, relaxation exercises) to overcome them. Module 5 teaches subjects how to employ metacognitive therapy to overcome ruminating thoughts. Finally, in module 6, participants reflect on strategies they tried during their training and their potential future application. In every session, participants are invited to plan recreational activities and to incorporate them into their daily life, as per the behavioral activation approach. In addition, at the end of each session, participants are asked to select and complete at least one exercise during the following week. The training included interactive exercises, audio/video files, and downloadable material and was presented on a secured Web-based platform. As training was based on self-help, participants received only technical support via email. Screenshots of the intervention are available in the Multimedia Appendix 1.

### Primary Outcome Measure

The study’s primary outcome was insomnia severity, measured using the German version of the Insomnia Severity Index (ISI; [49]). This questionnaire consists of 7 items, each answered on a 5-point Likert scale, with responses ranging from 0 to 4 (total range 0-28). Summation scores are categorized as follows [52]: absence of insomnia (0-7), subthreshold insomnia (8-14), moderate insomnia (15-21), and severe insomnia (22-28). The instrument is a widely recommended outcome measure for clinical studies on insomnia symptoms [53] and has been validated as a Web-based measure [54]. The ISI has demonstrated high internal consistency in both community and clinical samples, with an internal consistency between 0.90 and 0.91 [52].

### Secondary Outcome Measures

Secondary outcome measures assessed mental and work-related health and cognitive activities. Mental health outcomes included level of depression (Center for Epidemiological Studies-Depression Scale, consisting of 20 items with ratings ranging from 0 to 3; total range 0-60; alpha=.88; [55]) and recuperation in sleep (Recuperation in Sleep subscale, consisting of 8 items with ratings ranging from 1 to 5; alpha=.85; [56]). Work-related health outcomes included the frequency of recreational activities after work over the past week (Recreation Experience and Activity Questionnaire, consisting of 21 items with ratings ranging from 0 to 4; total range 0-84; alpha=.77; [57]) and work ability (single-item score from the Work Ability Index; range 0-10; [58]). To assess subjects’ self-rated number of full days on sick leave (absenteeism) and self-rated number of full days with reduced efficiency at work while feeling ill (presenteeism) over the past 3 months, the German Version of the Trimbos/Institute of Medical Technology Assessment questionnaire for costs associated with psychiatric illness was used [59], both at baseline and at 6-month follow-up. Cognitive activity was measured in 2 different ways: as work-related rumination (Cognitive Irritation subscale; 3 items ranging from 1 to 7; total range 3-28; alpha=.86; [60]) and as the subject’s general tendency to worry (Penn State Worry Questionnaire, Ultra Brief Version, past week; 3 items ranging from 0 to 6; total range 0-18; alpha=.85; [61]).

### Additional Measurements

Additional data collected included demographic variables and clients’ self-rated level of satisfaction with the intervention (Client Satisfaction Questionnaire, adapted to the online context; 8 items ranging from 0 to 4; total range 0-32, alpha=.93; [62,63]).

### Statistical Analyses

All analyses are reported in compliance with the Consolidated Standards of Reporting Trials of Electronic and Mobile HEalth Applications and onLine TeleHealth guidelines for improving and standardizing the report of Web-based and mobile health interventions [64] using intention-to-treat (ITT) procedures. Analyses were performed using IBM SPSS version 25 by a researcher who was not otherwise involved in the developing and conducting of the study. No interim analyses for intervention efficacy were conducted. Reported *P* values were 2-sided, with the a priori threshold for statistical significance set at .05.

#### Missing Data

All participants completed the baseline assessment. At 8 weeks (T2), 25% (n=33 in the iCBT-I group and n=11 in the control group) of the data were missing; at T3, 29% (n=42 and n=9 in the iCBT-I group and the control group, respectively) of the data were missing. Assumptions of normality were assessed graphically using histograms. The robustness of the assumption regarding missing outcome data was examined in a series of sensitivity analyses (missing data patterns and inclusion of sample characteristics associated with having missing outcomes, eg, number of completed modules). All existing data of the primary and secondary outcomes, the number of completed modules, the grouping variable, and the interaction term as a product of group allocation with baseline scores of insomnia were used in the imputation model [65]. Multiple imputations with 100 estimates per missing value were conducted to handle missing data [66]. We used predictive mean matching (PMM) to reduce the possible bias introduced in a dataset through imputation by drawing real values sampled from the observed data. The PMM method ensures that imputed values are plausible and is robust if the normality assumption is violated [66]. The iterative Markov Chain Monte Carlo method was used, as it is appropriate when the data have an arbitrary (monotone or nonmonotone) missing pattern [66].

#### Efficacy of Training

Analysis was performed in the ITT population, including all randomized patients, using a linear mixed effects model to account for the repeated measures at baseline, postintervention, and at 6-month follow-up. Fixed effects included group allocation, time (preintervention, postintervention, and 6-month follow-up), and the group-by-time interaction. Random effects were run to account for between-subject variation. Cohen *d* and corresponding 95% CIs were computed for T2 and T3, comparing the means and standard deviations for both the iCBT-I and control groups immediately postintervention and at 6-month follow-up. For this purpose, the standard deviation was calculated from the standard error [67].

#### Meaningful Improvement and Symptom-Free Status

To detect a meaningful improvement in insomnia severity from T1 to T2 and from T1 to T3, proposed change scores from a study by Morin et al [52] were obtained. On the basis of this, ISI score differences of more than 4.6 points—between T1 and either T2 or T3—were considered a slight improvement. This change score is approximately as high as the reliable change index of 5.01 points in the ISI used in previous studies on the present iCBT-I [27,28]. According to Morin et al [52], in an individual subject, change scores of more than 8.4 points were used to find a moderate improvement and change scores of more than 9.9 points were used to find a marked improvement. To detect potential negative effects of the intervention, the number of participants with meaningful symptom deterioration, according to the change scores above, was assessed. To assess symptom-free status, an ISI score below 8 was considered symptom free [52]. In addition, the number needed to treat (NNT) was calculated by comparing the 2 groups for the number of participants (1) with or without a meaningful improvement in insomnia severity and (2) who became or failed to become symptom free.

#### Mediators of Interventional Effects

To examine both forms of cognitive activity, measured as worry and work-related rumination, as potential mediators of interventional effects on the primary outcome at T2, parallel multiple mediation analysis was conducted using the PROCESS software for SPSS (model 4), with bias-corrected bootstraps based on 10,000 bootstrap samples [68]. To establish temporal precedence, the postintervention scores of the mediators and 6-month follow-up scores for the primary outcome were used. Following the recommendation of Valente and McKinnon [69], baseline scores for the mediating variables and primary outcome were included as covariates in the model. Statistical significance of the mediation was achieved if the estimated 95% CI for the indirect effect did not overlap zero [68].

#### Moderators of Interventional Effects

The study sample was heterogeneous with respect to insomnia severity at baseline, as there were no inclusion criteria regarding the severity of the latter. To assess whether the intervention’s effect on insomnia severity at postintervention and at 6-month follow-up is moderated by different levels of insomnia severity at baseline (before receiving the iCBT-I), simple moderation analysis was conducted using the PROCESS software for SPSS (model 1). For significant moderation, the Johnson-Neyman (J-N) procedure was employed [68,70] to identify the specific values of the moderator at which the groups differed significantly on the primary outcome at both assessment points.

## Results

### Participants

Figure 1 shows the flow of participants. In total, 177 individuals were randomized to iCBT-I (n=88) and control (n=89) groups. As the 10-month funding period that we were granted was inadequate to recruit the originally intended sample of 200 subjects and further recruitment was impossible, the final sample size was 177. With 177 subjects, the trial had 80% power to detect an intervention effect of *d*=0.42 at postintervention.

### Baseline Characteristics

Table 1 summarizes the baseline characteristics of study participants. The sample consisted of 177 workers, of whom 116 were female (116/177, 65.5%), and the average age was 46.4 years (SD 9.8). Most participants (141/177, 79.7%) were employed full time and had an average occupational experience of 20.7 years (SD 9.7). More than half of the study’s participants (103/177, 58.2%) had no previous experience with psychotherapy. Only 20 (20/177, 11.3%) participants had previously received psychotherapy for sleep problems, and just 33 (33/177, 18.6%) participants had undergone previous occupational health training. Most participants (123/177, 69.5%) reported clinically relevant insomnia symptoms (ISI score 15-28), whereas one-third of participants (54/177; 30.5%) had less or no insomnia symptoms (ISI score 0-14).

**Table 1:** Baseline characteristics.

Characteristics		Total (N=177)	Internet-delivered cognitive behavioral therapy for insomnia group (n=88)	Control group (n=89)
**Sociodemographics, n (%)**				
	Females	116 (65.5)	59 (67)	57 (64)
	Married/partnership	111 (62.7)	54 (61)	57 (64)
Age (years), mean (SD)		46.5 (9.8)	46.1 (9.5)	46.7 (9.7)
**Working characteristics**				
	Years of occupational experience, mean (SD)	20.7 (9.7)	20.4 (9.8)	21.1 (10.5)
	Permanent employment, n (%)	117 (66.1)	62 (71)	55 (62)
	Employed fulltime, n (%)	141 (79.7)	69 (78)	72 (81)
**Working sector, n (%)**				
	Health	35 (19.8)	15 (17)	20 (23)
	Economy	34 (19.2)	21 (24)	13 (15)
	Service	32 (18.1)	14 (16)	18 (20)
	Social	27 (15.3)	14 (16)	13 (15)
	Others	49 (27.7)	24 (27)	25 (28)
**Experiences with training or psychotherapy, n (%)**				
	Occupational mental health training	33 (18.6)	18 (21)	15 (17)
	Psychotherapy	74 (41.8)	34 (39)	40 (45)
	Psychotherapy for sleeping problems	20 (11.3)	7 (8)	13 (15)
**Insomnia severity, n (%)**				
	Severe (ISI^a^ score 22-28)	17 (9.6)	6 (7)	11 (12)
	Moderate (ISI score 15-21)	106 (59.9)	56 (64)	50 (56)
	Subthreshold (ISI score 8-14)	49 (27.7)	24 (27)	25 (28)
	Symptom free (ISI score 0-7)	5 (2.8)	2 (2)	3 (3)

^a^ISI: Insomnia Severity Index.

### Intervention Use and User Satisfaction

Of the 88 individuals who were assigned to the iCBT-I group, 17 (19%) dropped out before completing module 1 of the intervention. Module 1 was completed by 71 (71/88, 81%) participants, module 2 was completed by 56 (56/88, 64%) participants, module 3 was completed by 51 (51/88, 58%) participants, module 4 was completed by 46 (46/88, 52%) participants, module 5 was completed by 39 (39/88, 44%) participants, and all modules were completed by 35 (35/88, 40%) participants. On average, 3.4 modules (SD 2.3) were completed in the training group. Those who completed the training needed, on average, 73.8 days (SD 55.6). With regard to intervention completer, the total completion time of the entire training was, on average, 5.0 hours (SD 1.3). For all modules, most participants required, on average, 0.5 to 1 hour.

Only 3 participants reported their reason for dropout; stated reasons were *technical problems*, *lack of time*, and *already sufficient help before the last module*. Participants’ overall satisfaction with the training was high (n=54; mean 27.1 [SD 4.2]), with 93% (50/54) of the participants *satisfied in an overall, universal sense* (item 7, answering *generally yes* or *yes, completely*).

### Primary Outcome Analysis—Insomnia Severity

**Figure 2:**
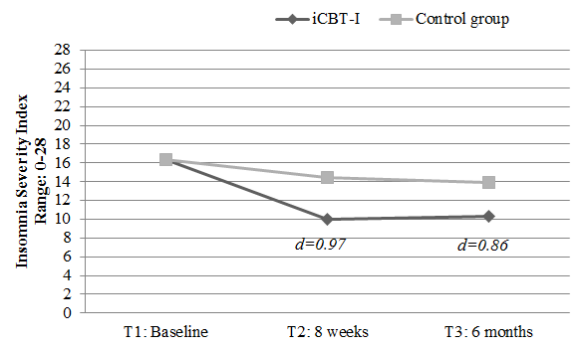
Comparison of internet-delivered cognitive behavioral therapy for insomnia and control groups on development of insomnia severity from baseline to 8 weeks after the training began and from baseline to 6-month follow-up. iCBT-I: internet-delivered cognitive behavioral therapy for insomnia.

For the primary outcome, the overall test of group-by-time interaction was significant (*P<*.001), with the intervention group reducing insomnia severity over time (Figure 2). Relative to controls, those who received the iCBT-I reported significantly reduced insomnia severity at T2 with a large effect size of Cohen *d*=0.97 and with a moderate-to-large effect size at 6-month follow-up (Cohen *d*=0.86). Table 2 displays all means, standard errors, and mean differences between groups for all outcome measures at T2 and T3 separately.

**Table 2:** Effects of internet-delivered cognitive behavioral therapy for insomnia group compared with the control group on primary and secondary outcomes.

Outcome			Internet-delivered cognitive behavioral therapy for insomnia group, mean (SE)	Control group, mean (SE)	Mean difference between groups (95% CI)	*P* value	Cohen *d* (95% CI)	Group × time, *P* value	
**Primary outcome**									
	**Insomnia Severity Index**								**<.001**
		T1^a^	16.35 (0.46)	16.32 (0.46)	N/A^b^	N/A	N/A		
		T2^c^	10.03 (0.48)	14.40 (0.48)	−4.36 (−5.69 to −3.03)	<.001	0.97 (0.66 to 1.28)		
		T3^d^	10.30 (0.45)	13.93 (0.45)	−3.64 (−4.89 to −2.39)	<.001	0.86 (0.55 to 1.17)		
**Mental health**									
	**Center for Epidemiological Studies-Depression Scale**								**<.001**
		T1	22.08 (0.89)	20.78 (0.88)	N/A	N/A	N/A		
		T2	15.40 (0.88)	17.91 (0.88)	−2.52 (−4.97 to −0.06)	.044	0.30 (0.01 to 0.60)		
		T3	16.75 (0.83)	20.30 (0.82)	−3.55 (−5.86 to −1.24)	.003	0.46 (0.16 to 0.76)		
	**Recuperation in Sleep subscale^e^**								**<.001**
		T1	2.35 (0.06)	2.43 (0.06)	N/A	N/A	N/A		
		T2	3.01 (0.07)	2.50 (0.07)	0.51 (0.31 to 0.70)	<.001	0.78 (0.47 to 1.08)		
		T3	3.11 (0.06)	2.58 (0.06)	0.53 (0.35 to 0.71)	<.001	0.94 (0.63 to 1.25)		
**Work-related health**									
	**Recreation Experience and Activity Questionnaire^e^**								**.121**
		T1	47.07 (1.02)	45.11 (1.01)	N/A	N/A	N/A		
		T2	51.4 (1.05)	46.65 (1.04)	4.75 (1.83 to 7.68)	.002	0.48 (0.18 to 078)		
		T3	51.10 (1.00)	47.12 (0.99)	3.98 (1.21 to 6.75)	.005	0.43 (0.13 to 0.72)		
	**Work Ability Index^e^**								**.006**
		T1	6.88 (0.18)	6.94 (0.17)	N/A	N/A	N/A		
		T3	7.14 (0.20)	6.46 (0.20)	0.68 (0.12 to 1.22)	.017	0.51 (0.21 to 0.81)		
	**Absenteeism^f^**								**.869**
		T1	4.62 (0.95)	2.69 (0.94)	N/A	N/A	N/A		
		T3	4.71 (1.14)	3.14 (1.14)	1.57 (−1.61 to 4.75)	.333	0.21 (−0.09 to 0.50)		
	**Presenteeism^f^**								**.103**
		T1	10.69 (1.43)	13.17 (1.42)	N/A	N/A	N/A		
		T3	5.94 (1.18)	12.39 (1.17)	−6.455 (−9.73 to 3.18)	<.001	0.83 (0.52 to 1.13)		
**Cognitive activity**									
	**Cognitive irritation subscale**								**<.001**
		T1	15.52 (0.37)	15.79 (0.36)	N/A	N/A	N/A		
		T2	11.18 (0.40)	14.43 (0.39)	−3.25 (−4.35 to −2.15)	<.001	0.87 (0.57 to 1.18)		
		T3	12.20 (0.39)	15.19 (0.39)	−2.99 (−4.08 to −1.90)	<.001	0.81 (0.51 to 1.12)		
	**Penn State Worry Questionnaire Past Week**								**<.001**
		T1	9.21 (0.41)	8.23 (0.41)	N/A	N/A	N/A		
		T2	5.77 (0.40)	6.85 (0.39)	−1.08 (−2.18 to 0.02)	.05	0.29 (−0.01 to 0.59)		
		T3	6.9 (0.39)	8.35 (0.39)	−1.46 (−2.55 to −0.36)	.009	0.39 (0.10 to 0.69)		

^a^At baseline.

^b^Not applicable.

^c^Postintervention (8 weeks postrandomization).

^d^At 6-month follow-up (4 months postintervention).

^e^Higher scores indicate better outcome.

^f^In relation to the previous 3 months.

### Meaningful Improvement and Symptom-Free Status

Meaningful improvements are divided into 3 categories according to Morin et al [52]. A change score of 4.6 points in ISI was classified as a slight improvement, a change score of 8.4 points was classified as a moderate improvement, and a change score of 9.9 points was classified as a marked improvement. Meaningful improvements in insomnia severity and corresponding numbers needed to treat for T2 and T3 are shown in Table 3.

At T2, more participants in the iCBT-I group (57/88, 65%) reported a slight meaningful improvement in insomnia severity than the participants in the control group (16/88, 18%), which yielded an NNT of 2.14 (95% CI 1.68 to 2.94) to achieve a meaningful improvement in insomnia severity (Δ4.6) from baseline to postintervention. In addition, at T3, more participants in the iCBT-I group reported a meaningful improvement in insomnia severity (60/88, 68%) than those in the control group (25/88, 28%), corresponding to an NNT of 2.49 (95% CI 1.87 to 3.76).

At T2, 1 iCBT-I group member and 2 control subjects experienced meaningful deterioration (T2 score of >4.6 above the T1 score). At 6-month follow-up, the corresponding numbers were 1 and 2. For the other 2 categories (moderate and marked), no deteriorations could be detected for T2 and T3.

Significantly, more iCBT-I group participants (20/88, 23%) were symptom free at T2 than those in the control group (8/88, 9%), corresponding to an NNT of 7.28 (95% CI 4.11 to 31.68). The same was true at T3, with significantly more iCBT-I group participants (16/88, 18%) than control group participants (6/88, 7%) claiming to be symptom free, generating an NNT of 8.74 (95% CI 4.75 to 54.21).

**Table 3:** Meaningful improvements in insomnia severity from baseline to 8 weeks after the training began and from baseline to 6-month follow-up.

Meaningful improvements in insomnia severity		Meaningful improvement		Number needed to treat (95% CI)	
		Internet-delivered cognitive behavioral therapy for insomnia group, n (%)	Control group, n (%)		
**From baseline to 8 weeks after the training began**					
	Slight	57 (65)	16 (18)		2.14 (1.68 to 2.94)
	Moderate	28 (32)	4 (4)		3.66 (2.63 to 5.99)
	Marked	20 (23)	3 (3)		5.17(3.46 to 10.17)
**From baseline to 6-month follow-up**					
	Slight	60 (68)	25 (28)		2.49 (1.87 to 3.76)
	Moderate	16 (18)	6 (7)		8.74 (4.75 to 54.21)
	Marked	75 (85)	86 (97)		8.87 (5.07 to 32.32)

### Secondary Outcome Analyses

As Table 2 shows, significant differences in favor of the iCBT-I group were evident at both assessment points for the mental health outcomes of depression and recuperation in sleep. Work-related health outcome was found to significantly differ between the 2 groups, with regard to recreational activities, presenteeism, and work ability at T2 and T3. However, the between-group difference of absenteeism was nonsignificant at T3 (*P*=.33). Relating to cognitive activity, significant differences between the 2 groups were identified for both measures of cognitive activity—work-related rumination and worry— at both assessment points. Effect sizes ranged from Cohen *d*=0.29 at T2 and Cohen *d*=0.39 at T3 for worry to Cohen *d*=0.87 at T2 and Cohen *d*=0.81 at T3 for work-related rumination.

### Mediators of Interventional Effects

**Figure 3:**
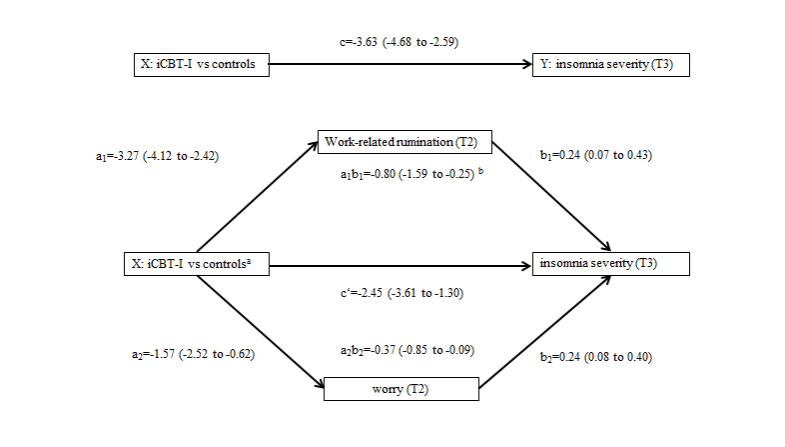
Parallel multiple mediation model with 6-month follow-up insomnia severity scores as the outcome variable, postintervention work-related rumination and worry scores as mediators, and baseline values of mediators and outcome as covariates. Interventiona X is coded 0=control groups and 1=iCBT-I group. bUnstandardized beta coefficients are shown, with 95% (bootstrapped biased corrected) CIs in parentheses. iCBT-I: internet-delivered cognitive behavioral therapy for insomnia.

As depicted in Figure 3, the results of parallel multiple mediation analysis indicated that both forms of cognitive activity—work-related rumination (indirect effect: a_1_b_1_=−0.80; SE 0.34; 95% boot CI −1.59 to −0.25) and worry (indirect effect: a_2_b_2_=−0.37; SE 0.19; 95% boot CI −0.85 to −0.09)—significantly mediated the effect of the intervention on insomnia severity at 6-month follow-up. The direct effect of the intervention to reduce insomnia remained significant after incorporating the 2 mediators in the model: (direct effect: *c'*=−2.45; SE 0.59; 95% CI −3.61 to −1.30). This indicates that the intervention also reduced insomnia, independent of its indirect effects on worry and work-related rumination.

### Moderators of Interventional Effects

Moderation analysis showed a significant group×baseline insomnia severity interaction (beta=−.68; SE 0.13; **t*_173_*=−5.37; *P*<.001; 95% CI −0.93 to −0.43) immediately postintervention, indicating that the intervention’s effect on insomnia severity was moderated by the severity of insomnia at baseline. Probing this effect, using the J-N technique, revealed a baseline ISI score of 12.08 as the point of transition between a statistically nonsignificant and significant effect of iCBT-I on postintervention insomnia severity. This means that, at T2, a significant group difference was identified for participants scoring 12 or greater on the baseline insomnia severity score (80% of all participants). No significant moderation effect of baseline levels of insomnia severity was detected for the intervention’s effect on insomnia severity at the 6-month follow-up assessment (beta=−.21; SE 0.12; **t*_173_*=−1.67; *P*=.10; 95% CI −0.45 to 0.04).

## Discussion

### Principal Findings

In this study, we examined the efficacy of a self-help Web-based recovery training to improve sleep among the general working population. Results showed evidence that iCBT-I adapted for workers could be effective in the working population up to 6 months after start of the intervention and even workers with less sleep problems at baseline benefitted from the training in the longer term. Furthermore, it has been demonstrated that in addition to general cognitive activity, work-related cognitive activity mediates the interventions effect on sleep.

So far, we are aware of only 2 other randomized controlled trials that have evaluated the efficacy of self-help iCBT-I in a general working population without insomnia severity as an inclusion criterion. These studies revealed moderate-to-large effects on sleep in the short term [29,30]. This study adds to the limited evidence for the efficacy of iCBT-I in the general working population, demonstrating that a self-help iCBT-I not only has short-term benefits with moderate-to-large effects but also has sustained benefits for up to 6 months. On average, participants changed from moderate insomnia to subthreshold category and managed to sustain their improvement. Even after 6 months, the NNT for a marked improvement (which represents a change score of about 10 points or more in insomnia severity) is still 8.87. Similar numbers were found in a recent meta-analysis of self-help intervention for depression at posttreatment [21]. Accordingly, 9 individuals are needed to achieve a marked improvement after 6 months, which represents a considerable effect taking the low costs of self-help iCBT-I and the context of universal prevention into account. The intervention’s moderate-to-large effects on insomnia severity are consistent with results from recent meta-analyses that mainly focused on iCBT-I in clinical samples or in indicated prevention setting [15-18]. The results were also comparable with moderate-to-large effects found for iCBT-I in samples of the working population with elevated insomnia symptoms as the inclusion criterion [24-26]. However, consistent with expectations, effects also were less than in previous studies evaluating the efficacy of this training when used as either a guided or self-guided version by a sample of teachers with elevated insomnia symptoms [27,28]. One possible explanation is that the training’s effects are greater in indicated prevention than in universal prevention settings, in which all interested workers could participate [45]. This might be explained by the fact that participants with raised levels of insomnia severity may be more motivated to implement what they were taught during the program and, therefore, produce more immediate postintervention results [45]. Another explanation might be found in the training’s self-guided mode of delivery, as guided internet-based mental health interventions have been observed to induce greater reductions in symptoms than those that are self-guided [71].

As this study was conducted in the general working population and all interested workers who were motivated to improve their sleep through an iCBT-I were included, the sample was more heterogeneous with regard to insomnia severity at baseline than those of previously published studies in the working population [15-18,24,26-28]. Approximately 30% of our study participants reported less or even no symptoms of insomnia (ISI ≤14), and all participants would have been excluded from previous studies that restricted recruitment to those with moderate-to-severe insomnia.

Moderation analysis revealed a significant moderation effect of baseline insomnia severity level postintervention. At 6-month follow-up, no moderation effect was identified, indicating that participants’ level of insomnia severity at baseline did not influence the intervention’s efficacy over the long term. These findings are important because they indicate that a substantial portion of the working population—those reporting clinical relevant insomnia with an ISI score of 12 or more—were benefitting from the intervention immediately after the training program ended and that the training was even effective for participants with less baseline insomnia severity scores in the long term. This could be interpreted as a support for the idea of prevention. Those workers who face only less severe sleep complaints at baseline might benefit from participating in the interventions in the longer term, as they might equip themselves with skills that will help them to prevent that less severe symptoms turn not into more severe symptoms over time. In turn, workers without access to the intervention with less severe insomnia at baseline could develop more severe insomnia over the long term [72]. Considering that untreated subclinical insomnia can progress into clinical insomnia [11,72], in turn, potentially adversely affecting long-term health [9-11], the currently tested training might be a useful and effective measure for the prevention of more severe insomnia. Furthermore, the participation rate of 30.5% workers with no or subthreshold insomnia severity yields initial insight into the training’s potential reach for workers with less sleep complaints in routine occupational health care.

It is notable that the intervention also was effective at reducing depressive symptoms, underlining the close relationship between sleep and depression [8]. These effects were even greater in magnitude at 6-month follow-up. In previously reported studies evaluating CBT-I, similar effects have been observed in those with moderately severe depressive symptoms, indicating that improvements in mood continued after intervention [73,74]. Considering these results, iCBT-I appears to be beneficial for both sleep and depression [8,16].

Apart from its positive effects on sleep and depression, the training we tested also exhibited meaningful effects on work-related health outcomes. Participants in the intervention group changed their health behaviors, indicating more frequent participation in positive recreational activities after work when the program ended and maintaining their healthy behavior change through 6 months. Similar effects on recreational activities were detected in 2 recent studies in teachers [27,28].

We identified mixed effects for work productivity, however. On the one hand, no significant effect on absenteeism was identified. This might be explained by a floor effect, as absenteeism was generally very low in the present sample. On the other hand, a significant, moderate effect was identified for presenteeism. This is also consistent with previously published findings, which indicate that insomnia primarily affects presenteeism, while it affects absenteeism only to a smaller extent [27,28]. It is also supported by health economics research that has documented how the positive economic effects of iCBT-I are mainly attributable to its influence on presenteeism [20]. Furthermore, the intervention affected subjects’ ratings of their actual ability to work. Participants in the intervention group reported significantly higher work ability than those in the control group. These results are notable as workers suffering from sleep complaints often show poorer work performance [31].

Cognitive models of insomnia highlight the important role that negatively toned presleep cognitive activity plays in the development and persistence of insomnia [32,37]. For this reason, the present intervention also focused on reducing presleep cognitive activity, thereby considering both general and work-related cognitive activities. Small effects were identified in terms of reducing general cognitive activity and measured as worry. Conversely, a reduction in work-related cognitive activity, in terms of work-related rumination, showed large effects. These effects are important because work-related rumination has been shown to be a mediator between work-stress and impaired sleep [33], and both rumination [75] and insomnia [7] are predictors of future depression.

Despite the extensive literature citing cognitive activity as a key developmental and maintenance factor for insomnia [32,37], little is known about how iCBT-I improves sleep by reducing presleep cognitive activity [28,36,43,76]. Our secondary aim was to exploratory examine whether, in addition to the role of general cognitive activity, work-related cognitive activity might be a mediator of the intervention’s efficacy. Results of parallel multiple mediation analyses help to clarify such mechanisms, revealing that our intervention’s effect on sleep was mediated by reducing the following 2 forms of cognitive activity that we studied: worry and work-related rumination. In other words, in our sample, the training indirectly enhanced sleep by reducing general worry and work-related rumination. This suggests that tailoring an intervention to the specific needs of workers to reduce work-related cognitive activity can be a promising approach to effectively reduce sleep problems of workers. This further supports prior research documenting the benefits of tailoring interventions to users’ life domains [77].

To summarize, our results suggest that reducing presleep cognitive activity could be an important component of any effective intervention for insomnia, and that including components that target reducing work-related cognitive activity might be an important contribution to designing interventions that reduce sleep complaints in workers.

### Limitations

The following limitations of this trial must be acknowledged. First, because of the restricted funding period, the final sample size differed from the sample size initially intended. However, given the intervention’s sizeable effect on the primary outcome, we believe that our conclusions are not substantially affected by the smaller sample size. Second, the intervention was not offered within a typical workplace setting (eg, providing the intervention within the company or informing about participation options within the confines of employee assemblies or other in-house communication). The recruitment strategy used in this study (ie, mass media and email distribution list from one of the largest health insurance companies in Germany) presents a complementary way of approaching workers who are interested in improving their sleep. Therefore, it is unclear if our results can be generalized to routine occupational health care settings approaching workers within the company. Nonetheless, the applied recruiting strategy here mimics 1 possibility of how internet-delivered mental health interventions are made available for workers in Germany.

Third, we used a waitlist-control and no attention control group. Thus, it is not possible to determine whether and to what extent the effects of the intervention were because of specific iCBT-I content or because of nonspecific support provided by participating in an intervention. Moreover, one might expect that the delayed access to the training for the control group leads to a sense of disappointment and rumination about sleep while waiting to receive the intervention. Measuring disappointment would have been a way of controlling such sources of bias, which should be considered in further studies with waitlist-control groups. Moreover, in future studies, it would be of practical importance to employ other self-help interventions as a comparator (ie, self-help books to determine the unique contribution of the internet-delivered format) [78]. However, it should be mentioned that previous studies largely used similar control groups; therefore, the results of this study can be compared with existing research [24,27-30].

Fourth, there was a high dropout rate as defined by the study protocol and by the completion of modules among participants in the intervention group. This could lead to an overestimation of the intervention effect because it is assumed that participants who were not satisfied with the intervention nor appeared to have experienced any benefit may not have answer any further questionnaires postintervention and at 6-month follow-up [79]. Thus, these participants may not have provided data. On the other hand, these participants could also be early completer who benefit from the intervention but do not complete the study protocol and finish the intervention early [80]. In addition, to reduce a biased estimate of the missing data, the number of modules completed was included in the imputation model [65].

Fifth, although no inclusion criteria with regard to insomnia severity were set up in context of universal prevention, approximately 60% of participants had moderate insomnia symptoms, and approximately 30% had subthreshold insomnia symptoms or were symptom free. As recruiting participants in the general working population for this study overlapped timewise with another study, it is likely that the percentage of workers with severe insomnia symptoms might be underestimated. This said, the results of this study are the first indication of the acceptance and efficacy of iCBT-I in a universal prevention setting in routine occupational health care and demonstrate that a substantial proportion of workers with no or less sleep problems are motivated to participate.

Finally, no measures were assessed to determine which of the training elements were the most effective and which elements were less effective. However, results from mediation analysis suggest that techniques that reduce presleep cognitive activity played an important role in the training’s efficacy. To explore which of the intervention’s components work best, further research is needed.

### Conclusions

In conclusion, the results of this study give further indications that an internet-delivered self-help CBT-I adapted for workers has stable effects up to 6 months after the training began.

Moreover, the presented training was open to all workers and a substantial proportion with less sleep problems participated, who benefitted from the training in the longer term. In this way, the present findings strengthen the arguments as to the generalizability and robustness of prior results on this training’s efficacy [27,28] and also that of prior results of iCBT-I in the working population [24-26,29,30]. The intervention’s substantial effect is noteworthy, as the training was delivered in a self-help form that increases its potential for large-scale and cost-effective implementation in universal prevention. Conclusively, from a public health perspective, more workers who are potentially in need of an intervention to improve their sleep could be reached.

## Appendix

Multimedia Appendix 1 Screenshots of the intervention.

Multimedia Appendix 2 CONSORT‐EHEALTH checklist (V 1.6.1).

## Abbreviations

CBT-I: cognitive behavioral therapy for insomnia
DRKS: German Clinical Trials Register
iCBT-I: internet-delivered cognitive behavioral therapy for insomnia
ISI: Insomnia Severity Index
ITT: intention-to-treat
J-N: Johnson-Neyman
NNT: number needed to treat
PMM: predictive mean matching
