# Supplementary material for: Efficacy of a Self-Help Web-Based Recovery Training in Improving Sleep in Workers: Randomized Controlled Trial in the General Working Population
Source: J Med Internet Res. 2020 Jan 7;22(1):e13346. doi: 10.2196/13346 (PMC6996739; doi:10.2196/13346)
Supplement: Multimedia Appendix 1 [file jmir_v22i1e13346_app1.pdf]

Supplemental material

Screenshots of the intervention

Picture 1: Starting page of the training

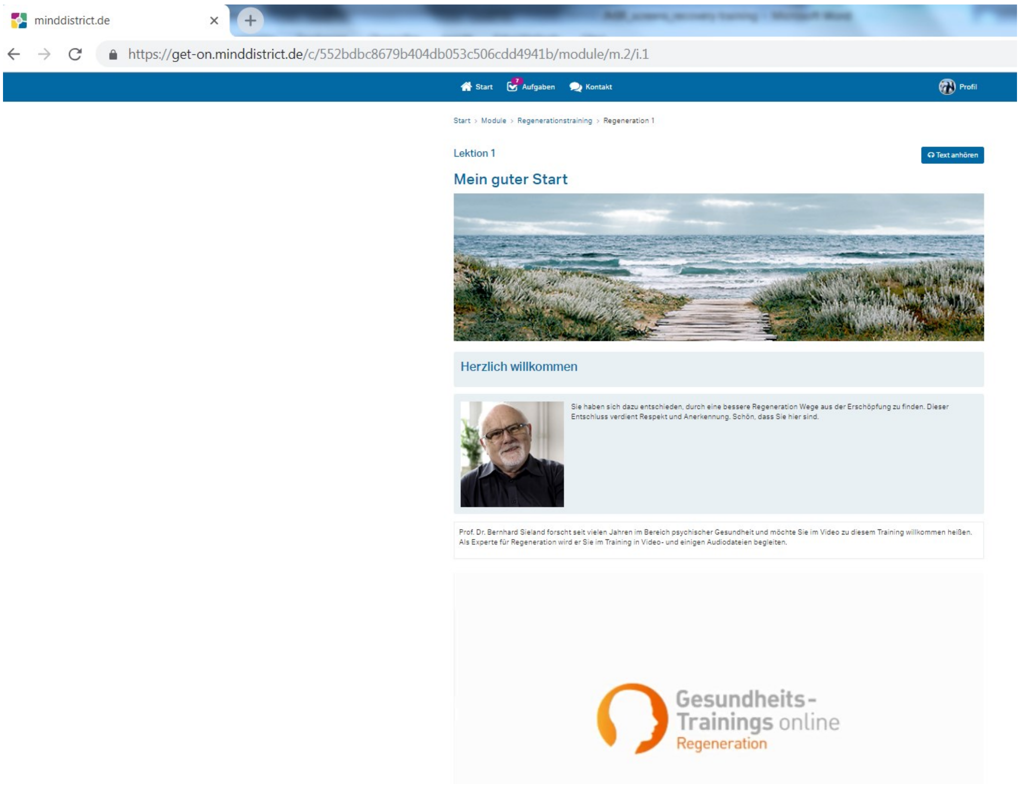

Picture 2: psychoeducation on healthy sleep

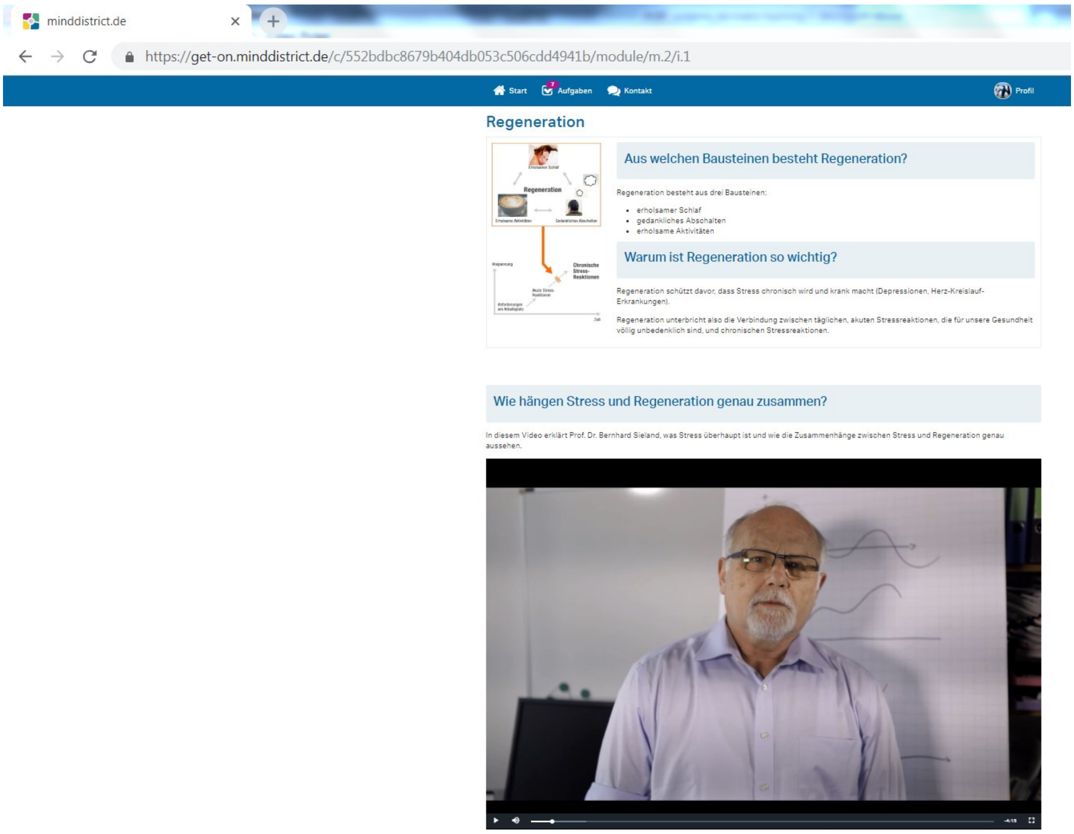

Picture 3: boundary tactics to support mental detachment from work

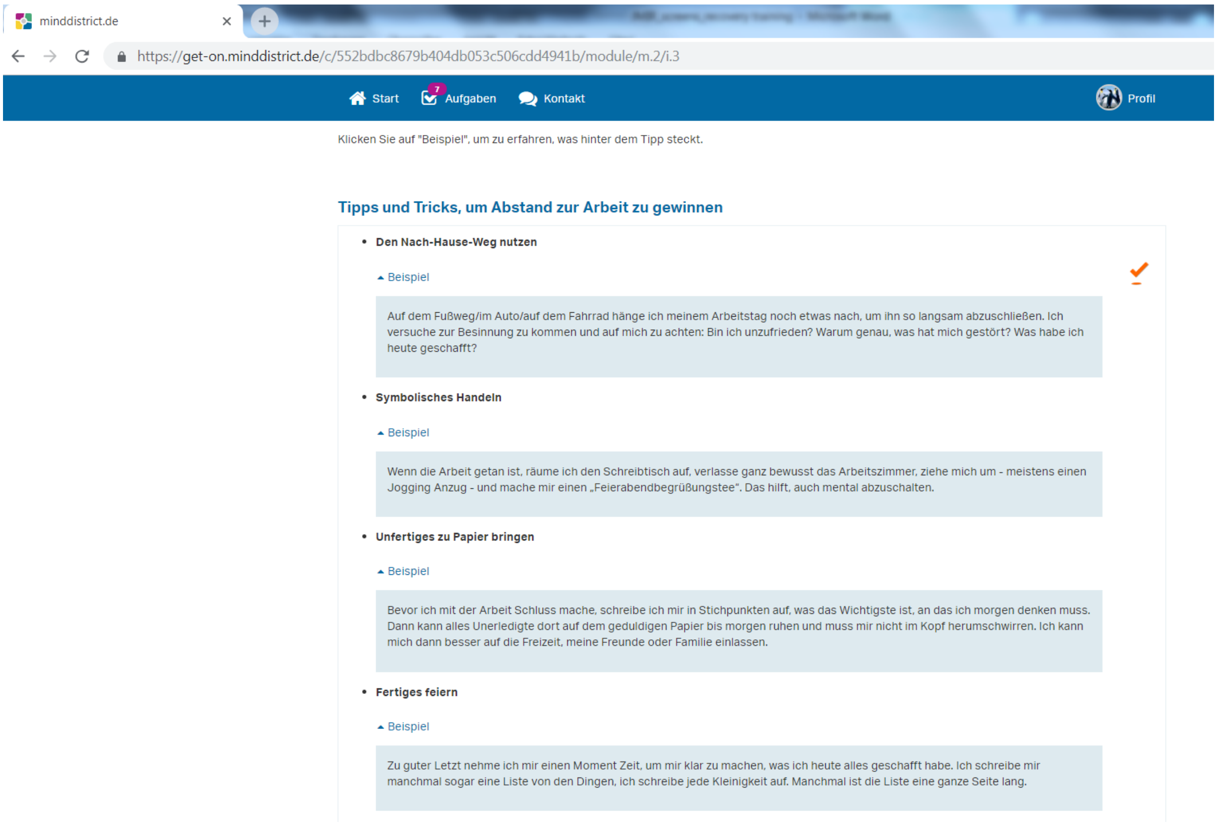
minddistrict.de

https://get-on.minddistrict.de/c/552bdbc8679b404db053c506cdd4941b/module/m.2/i.3

Start | Aufgaben | Kontakt | Profil

Klicken Sie auf "Beispiel", um zu erfahren, was hinter dem Tipp steckt.

Tipps und Tricks, um Abstand zur Arbeit zu gewinnen

- **Den Nach-Hause-Weg nutzen**

  Beispiel

  Auf dem Fußweg/im Auto/auf dem Fahrrad hänge ich meinem Arbeitstag noch etwas nach, um ihn so langsam abzuschließen. Ich versuche zur Besinnung zu kommen und auf mich zu achten: Bin ich unzufrieden? Warum genau, was hat mich gestört? Was habe ich heute geschafft?

- **Symbolisches Handeln**

  Beispiel

  Wenn die Arbeit getan ist, räume ich den Schreibtisch auf, verlasse ganz bewusst das Arbeitszimmer, ziehe mich um - meistens einen Jogging Anzug - und mache mir einen „Feierabendbegrüßungstee". Das hilft, auch mental abzuschalten.

- **Unfertiges zu Papier bringen**

  Beispiel

  Bevor ich mit der Arbeit Schluss mache, schreibe ich mir in Stichpunkten auf, was das Wichtigste ist, an das ich morgen denken muss. Dann kann alles Unerledigte dort auf dem geduldigen Papier bis morgen ruhen und muss mir nicht im Kopf herumschwirren. Ich kann mich dann besser auf die Freizeit, meine Freunde oder Familie einlassen.

- **Fertiges feiern**

  Beispiel

  Zu guter Letzt nehme ich mir einen Moment Zeit, um mir klar zu machen, was ich heute alles geschafft habe. Ich schreibe mir manchmal sogar eine Liste von den Dingen, ich schreibe jede Kleinigkeit auf. Manchmal ist die Liste eine ganze Seite lang.
