# Supplementary material for: Efficacy of a Self-Help Web-Based Recovery Training in Improving Sleep in Workers: Randomized Controlled Trial in the General Working Population
Source: J Med Internet Res. 2020 Jan 7;22(1):e13346. doi: 10.2196/13346 (PMC6996739; doi:10.2196/13346)
Supplement: Multimedia Appendix 2 [file jmir_v22i1e13346_app2.pdf]

CONSORT-EHEALTH (V 1.6.1) - Submission/Publication Form

The CONSORT-EHEALTH checklist is intended for authors of randomized trials evaluating web-based and Internet-based applications/interventions, including mobile interventions, electronic games (incl multiplayer games), social media, certain telehealth applications, and other interactive and/or networked electronic applications. Some of the items (e.g. all subitems under item 5 - description of the intervention) may also be applicable for other study designs.

The goal of the CONSORT EHEALTH checklist and guideline is to be
a) a guide for reporting for authors of RCTs,
b) to form a basis for appraisal of an ehealth trial (in terms of validity)

CONSORT-EHEALTH items/subitems are MANDATORY reporting items for studies published in the Journal of Medical Internet Research and other journals / scientific societies endorsing the checklist.

Items numbered 1., 2., 3., 4a., 4b etc are original CONSORT or CONSORT-NPT (non-pharmacologic treatment) items.
Items with Roman numerals (i., ii, iii, iv etc.) are CONSORT-EHEALTH extensions/clarifications.

As the CONSORT-EHEALTH checklist is still considered in a formative stage, we would ask that you also RATE ON A SCALE OF 1-5 how important/useful you feel each item is FOR THE PURPOSE OF THE CHECKLIST and reporting guideline (optional).

Mandatory reporting items are marked with a red *.
In the textboxes, either copy & paste the relevant sections from your manuscript into this form - please include any quotes from your manuscript in QUOTATION MARKS,
or answer directly by providing additional information not in the manuscript, or elaborating on why the item was not relevant for this study.

YOUR ANSWERS WILL BE PUBLISHED AS A SUPPLEMENTARY FILE TO YOUR PUBLICATION IN JMIR AND ARE CONSIDERED PART OF YOUR PUBLICATION (IF ACCEPTED).
Please fill in these questions diligently. Information will not be copyedited, so please use proper spelling and grammar, use correct capitalization, and avoid abbreviations.

DO NOT FORGET TO SAVE AS PDF _AND_ CLICK THE SUBMIT BUTTON SO YOUR ANSWERS ARE IN OUR DATABASE !!!

Citation Suggestion (if you append the pdf as Appendix we suggest to cite this paper in the caption):
Eysenbach G, CONSORT-EHEALTH Group
CONSORT-EHEALTH: Improving and Standardizing Evaluation Reports of Web-based and Mobile Health Interventions
J Med Internet Res 2011;13(4):e126
URL: http://www.jmir.org/2011/4/e126/
doi: 10.2196/jmir.1923
PMID: 22209829

* Erforderlich

Your name *

First Last

Doerte Behrendt

Primary Affiliation (short), City, Country *

University of Toronto, Toronto, Canada

Leuphana University Lueneburg

Your e-mail address *

abc@gmail.com

behrendt@leuphana.de

Title of your manuscript *

Provide the (draft) title of your manuscript.

Efficacy of a self-help web-based recovery training improving sleep in workers: results of a randomized controlled trial in universal prevention

Name of your App/Software/Intervention *

If there is a short and a long/alternate name, write the short name first and add the long name in brackets.

GET.ON RECOVERY

Evaluated Version (if any)

e.g. "V1", "Release 2017-03-01", "Version 2.0.27913"

Meine Antwort

Language(s) *

What language is the intervention/app in? If multiple languages are available, separate by comma (e.g. "English, French")

German

URL of your Intervention Website or App

e.g. a direct link to the mobile app on app in appstore (itunes, Google Play), or URL of the website. If the intervention is a DVD or hardware, you can also link to an Amazon page.

Meine Antwort

URL of an image/screenshot (optional)

https://get-on.minddistrict.de/

Accessibility *

Can an enduser access the intervention presently?

- ○ access is free and open
- ○ access only for special usergroups, not open
- ○ access is open to everyone, but requires payment/subscription/in-app purchases
- ○ app/intervention no longer accessible
- ◉ Sonstiges: is available free of charge for study participants, costs are incu[illegible]

Primary Medical Indication/Disease/Condition *

e.g. "Stress", "Diabetes", or define the target group in brackets after the condition, e.g. "Autism (Parents of children with)", "Alzheimers (Informal Caregivers of)"

insomnia

Primary Outcomes measured in trial *

comma-separated list of primary outcomes reported in the trial

insomnia severity index [ISI]

Secondary/other outcomes

Are there any other outcomes the intervention is expected to affect?

Center for Epidemiological Studies' Depression Scale [CES-D]; recuperation in sleep subscale [SF-B]; Recreation Experience and Activity Questionnaire [ReaQ]; work ability (single-item score from the Work Ability Index [WAI]; absenteeism and presenteeism over the past three months, German Version of the Trimbos/iMTA questionnaire for costs associated with psychiatric illness (TiC-P-G); Cognitive Irritation subscale [IS]; Penn State Worry Questionnaire, Ultra Brief Version, past week, [PSWQ-PW]; Client Satisfaction Questionnaire [CSQ-8]

Recommended "Dose" *

What do the instructions for users say on how often the app should be used?

- ○ Approximately Daily
- ◉ Approximately Weekly
- ○ Approximately Monthly
- ○ Approximately Yearly
- ○ "as needed"
- ○ Sonstiges:

Approx. Percentage of Users (starters) still using the app as recommended after 3 months *

- (x) unknown / not evaluated
- ( ) 0-10%
- ( ) 11-20%
- ( ) 21-30%
- ( ) 31-40%
- ( ) 41-50%
- ( ) 51-60%
- ( ) 61-70%
- ( ) 71%-80%
- ( ) 81-90%
- ( ) 91-100%
- ( ) Sonstiges:

Overall, was the app/intervention effective? *

- (x) yes: all primary outcomes were significantly better in intervention group vs control
- ( ) partly: SOME primary outcomes were significantly better in intervention group vs control
- ( ) no statistically significant difference between control and intervention
- ( ) potentially harmful: control was significantly better than intervention in one or more outcomes
- ( ) inconclusive: more research is needed
- ( ) Sonstiges:

Article Preparation Status/Stage *

At which stage in your article preparation are you currently (at the time you fill in this form)

- ○ not submitted yet - in early draft status
- ◉ not submitted yet - in late draft status, just before submission
- ○ submitted to a journal but not reviewed yet
- ○ submitted to a journal and after receiving initial reviewer comments
- ○ submitted to a journal and accepted, but not published yet
- ○ published
- ○ Sonstiges:

Journal *

If you already know where you will submit this paper (or if it is already submitted), please provide the journal name (if it is not JMIR, provide the journal name under "other")

- ○ not submitted yet / unclear where I will submit this
- ◉ Journal of Medical Internet Research (JMIR)
- ○ JMIR mHealth and UHealth
- ○ JMIR Serious Games
- ○ JMIR Mental Health
- ○ JMIR Public Health
- ○ JMIR Formative Research
- ○ Other JMIR sister journal
- ○ Sonstiges:

Is this a full powered effectiveness trial or a pilot/feasibility trial? *

- ○ Pilot/feasibility
- ◉ Fully powered

Manuscript tracking number *

If this is a JMIR submission, please provide the manuscript tracking number under "other" (The ms tracking number can be found in the submission acknowledgement email, or when you login as author in JMIR. If the paper is already published in JMIR, then the ms tracking number is the four-digit number at the end of the DOI, to be found at the bottom of each published article in JMIR)

- (x) no ms number (yet) / not (yet) submitted to / published in JMIR
- ( ) Sonstiges:

TITLE AND ABSTRACT

1a) TITLE: Identification as a randomized trial in the title

1a) Does your paper address CONSORT item 1a? *

I.e does the title contain the phrase "Randomized Controlled Trial"? (if not, explain the reason under "other")

- (x) yes
- ( ) Sonstiges:

1a-i) Identify the mode of delivery in the title

Identify the mode of delivery. Preferably use “web-based” and/or “mobile” and/or “electronic game” in the title. Avoid ambiguous terms like “online”, “virtual”, “interactive”. Use “Internet-based” only if Intervention includes non-web-based Internet components (e.g. email), use “computer-based” or “electronic” only if offline products are used. Use “virtual” only in the context of “virtual reality” (3-D worlds). Use “online” only in the context of “online support groups”. Complement or substitute product names with broader terms for the class of products (such as “mobile” or “smart phone” instead of “iphone”), especially if the application runs on different platforms.

	1	2	3	4	5	
subitem not at all important	( )	( )	( )	( )	( )	essential

Does your paper address subitem 1a-i? *

Copy and paste relevant sections from manuscript title (include quotes in quotation marks "like this" to indicate direct quotes from your manuscript), or elaborate on this item by providing additional information not in the ms, or briefly explain why the item is not applicable/relevant for your study

"Efficacy of a self-help web-based recovery training improving sleep in workers: results of a randomized controlled trial in universal prevention"

1a-ii) Non-web-based components or important co-interventions in title

Mention non-web-based components or important co-interventions in title, if any (e.g., “with telephone support”).

	1	2	3	4	5	
subitem not at all important	○	○	○	○	○	essential

Does your paper address subitem 1a-ii?

Copy and paste relevant sections from manuscript title (include quotes in quotation marks "like this" to indicate direct quotes from your manuscript), or elaborate on this item by providing additional information not in the ms, or briefly explain why the item is not applicable/relevant for your study

There are no non web-based components.

1a-iii) Primary condition or target group in the title

Mention primary condition or target group in the title, if any (e.g., “for children with Type I Diabetes”) Example: A Web-based and Mobile Intervention with Telephone Support for Children with Type I Diabetes: Randomized Controlled Trial

	1	2	3	4	5	
subitem not at all important	○	○	○	○	○	essential

Does your paper address subitem 1a-iii? *

Copy and paste relevant sections from manuscript title (include quotes in quotation marks "like this" to indicate direct quotes from your manuscript), or elaborate on this item by providing additional information not in the ms, or briefly explain why the item is not applicable/relevant for your study

"in workers"

1b) ABSTRACT: Structured summary of trial design, methods, results, and conclusions

NPT extension: Description of experimental treatment, comparator, care providers, centers, and blinding status.

1b-i) Key features/functionalities/components of the intervention and comparator in the METHODS section of the ABSTRACT

Mention key features/functionalities/components of the intervention and comparator in the abstract. If possible, also mention theories and principles used for designing the site. Keep in mind the needs of systematic reviewers and indexers by including important synonyms. (Note: Only report in the abstract what the main paper is reporting. If this information is missing from the main body of text, consider adding it)

	1	2	3	4	5	
subitem not at all important	○	○	○	○	○	essential

Does your paper address subitem 1b-i? *

Copy and paste relevant sections from the manuscript abstract (include quotes in quotation marks "like this" to indicate direct quotes from your manuscript), or elaborate on this item by providing additional information not in the ms, or briefly explain why the item is not applicable/relevant for your study

"A sample of 177 workers was randomized to either the iCBT-I (n = 88) or to care as usual (n = 89). The intervention is a 6-week web-based training consisting of 6 one-week modules."

1b-ii) Level of human involvement in the METHODS section of the ABSTRACT

Clarify the level of human involvement in the abstract, e.g., use phrases like “fully automated” vs. “therapist/nurse/care provider/physician-assisted” (mention number and expertise of providers involved, if any). (Note: Only report in the abstract what the main paper is reporting. If this information is missing from the main body of text, consider adding it)

	1	2	3	4	5	
subitem not at all important	○	○	○	○	○	essential

Does your paper address subitem 1b-ii?

Copy and paste relevant sections from the manuscript abstract (include quotes in quotation marks "like this" to indicate direct quotes from your manuscript), or elaborate on this item by providing additional information not in the ms, or briefly explain why the item is not applicable/relevant for your study

"Since the training was conducted in self-help, participants received nothing but technical support via email."

1b-iii) Open vs. closed, web-based (self-assessment) vs. face-to-face assessments in the METHODS section of the ABSTRACT

Mention how participants were recruited (online vs. offline), e.g., from an open access website or from a clinic or a closed online user group (closed usergroup trial), and clarify if this was a purely web-based trial, or there were face-to-face components (as part of the intervention or for assessment). Clearly say if outcomes were self-assessed through questionnaires (as common in web-based trials). Note: In traditional offline trials, an open trial (open-label trial) is a type of clinical trial in which both the researchers and participants know which treatment is being administered. To avoid confusion, use “blinded” or “unblinded” to indicated the level of blinding instead of “open”, as “open” in web-based trials usually refers to “open access” (i.e. participants can self-enrol). (Note: Only report in the abstract what the main paper is reporting. If this information is missing from the main body of text, consider adding it)

	1	2	3	4	5	
subitem not at all important	○	○	○	○	○	essential

Does your paper address subitem 1b-iii?

Copy and paste relevant sections from the manuscript abstract (include quotes in quotation marks "like this" to indicate direct quotes from your manuscript), or elaborate on this item by providing additional information not in the ms, or briefly explain why the item is not applicable/relevant for your study

"Web-based self-report assessments were scheduled at baseline, at eight weeks and six months following randomization."

1b-iv) RESULTS section in abstract must contain use data

Report number of participants enrolled/assessed in each group, the use/uptake of the intervention (e.g., attrition/adherence metrics, use over time, number of logins etc.), in addition to primary/secondary outcomes. (Note: Only report in the abstract what the main paper is reporting. If this information is missing from the main body of text, consider adding it)

	1	2	3	4	5	
subitem not at all important	○	○	○	○	○	essential

Does your paper address subitem 1b-iv?

Copy and paste relevant sections from the manuscript abstract (include quotes in quotation marks "like this" to indicate direct quotes from your manuscript), or elaborate on this item by providing additional information not in the ms, or briefly explain why the item is not applicable/relevant for your study

"An intention-to-treat analysis of covariance showed that, relative to controls, treated subjects reported significantly lower insomnia severity scores, at post treatment (F(1,174) = 64.26, P < .001; Cohen´s d = 0.97, 95% CI 0.66 – 1.28), and at six-month follow-up (F(1,174) = 49.19, P < .001; Cohen´s d = 0.83, 95% CI 0.52 – 1.14). Significant differences, with small-to-large effect sizes, also were detected for cognitive activation, and for mental and work-related health, but not for absenteeism. Mediation analysis demonstrated that work-related rumination (indirect effect : b= -0.99, SE =.35, 95% Boot CI -1.80 – -0.41) and worry (indirect effect: b = -0.26, SE = 0.35, 95% Boot CI -0.68 – -0.04) mediate the intervention's effect on sleep, the indirect effect being larger through work-related rumination than worry."

1b-v) CONCLUSIONS/DISCUSSION in abstract for negative trials

Conclusions/Discussions in abstract for negative trials: Discuss the primary outcome - if the trial is negative (primary outcome not changed), and the intervention was not used, discuss whether negative results are attributable to lack of uptake and discuss reasons. (Note: Only report in the abstract what the main paper is reporting. If this information is missing from the main body of text, consider adding it)

	1	2	3	4	5	
subitem not at all important	○	○	○	○	○	essential

Does your paper address subitem 1b-v?

Copy and paste relevant sections from the manuscript abstract (include quotes in quotation marks "like this" to indicate direct quotes from your manuscript), or elaborate on this item by providing additional information not in the ms, or briefly explain why the item is not applicable/relevant for your study

Not applicable

INTRODUCTION

2a) In INTRODUCTION: Scientific background and explanation of rationale

2a-i) Problem and the type of system/solution

Describe the problem and the type of system/solution that is object of the study: intended as stand-alone intervention vs. incorporated in broader health care program? Intended for a particular patient population? Goals of the intervention, e.g., being more cost-effective to other interventions, replace or complement other solutions? (Note: Details about the intervention are provided in “Methods” under 5)

	1	2	3	4	5	
subitem not at all important	○	○	○	○	○	essential

Does your paper address subitem 2a-i? *

Copy and paste relevant sections from the manuscript (include quotes in quotation marks "like this" to indicate direct quotes from your manuscript), or elaborate on this item by providing additional information not in the ms, or briefly explain why the item is not applicable/relevant for your study

"Impaired sleep is a common complaint and among the most prevalent health concerns. In Western industrialized countries, approximately 30-35% of the general adult population report insomnia symptoms, and approximately 10% meet the criteria of insomnia as a disorder [1]. A similar situation exists in the German working population, amongst whom one study documented a 4-week prevalence of 9.4% for insomnia disorder [2]. In the same study, an additional 35.1% of the working population reported subclinical symptoms of insomnia [2]."
"Published guidelines recommend insomnia-specific cognitive behavioural therapy (CBT-I) as first-line treatment [24], as it has been shown to produce large, sustainable effects [25]."
"Recent meta-analyses involving clinical samples have demonstrated that internet-delivered CBT-I (iCBT-I) is an effective alternative to face-to-face CBT-I [27–30]."
"This said, most previous studies on iCBT-I were not designed to reach the general working population, either targeting clinical samples or conducted in an indicated prevention setting, thereby excluding a substantial portion of the population with subclinical insomnia symptoms [27–30]. Consequently, results from previously-published studies on iCBT-I might not be broadly generalizable to the general working population. "

2a-ii) Scientific background, rationale: What is known about the (type of) system

Scientific background, rationale: What is known about the (type of) system that is the object of the study (be sure to discuss the use of similar systems for other conditions/diagnoses, if appropiate), motivation for the study, i.e. what are the reasons for and what is the context for this specific study, from which stakeholder viewpoint is the study performed, potential impact of findings [2]. Briefly justify the choice of the comparator.

	1	2	3	4	5	
subitem not at all important	○	○	○	○	○	essential

Does your paper address subitem 2a-ii? *

Copy and paste relevant sections from the manuscript (include quotes in quotation marks "like this" to indicate direct quotes from your manuscript), or elaborate on this item by providing additional information not in the ms, or briefly explain why the item is not applicable/relevant for your study

"Recent meta-analyses involving clinical samples have demonstrated that internet-delivered CBT-I (iCBT-I) is an effective alternative to face-to-face CBT-I [27–30]. Moreover, iCBT-I has been shown to improve not only insomnia, but other mental health outcomes, including symptoms of depression [31]."

2b) In INTRODUCTION: Specific objectives or hypotheses

Does your paper address CONSORT subitem 2b? *

Copy and paste relevant sections from the manuscript (include quotes in quotation marks "like this" to indicate direct quotes from your manuscript), or elaborate on this item by providing additional information not in the ms, or briefly explain why the item is not applicable/relevant for your study

"The primary aim of the current study was (1) to investigate the efficacy of the self-help version of GET.ON Recovery as a universal-prevention tool, employing both short- and long-term assessments of treatment effects. We also examined (2) whether previous results on this training´s efficacy are generalizable to the general working population; and (3) whether, in addition to the role of general cognitive activation, work-related cognitive activation is a putative mediator of the intervention’s efficacy."

METHODS

3a) Description of trial design (such as parallel, factorial) including allocation ratio

Does your paper address CONSORT subitem 3a? *

Copy and paste relevant sections from the manuscript (include quotes in quotation marks "like this" to indicate direct quotes from your manuscript), or elaborate on this item by providing additional information not in the ms, or briefly explain why the item is not applicable/relevant for your study

"[...] after which they were randomized to one of the two treatment arms, using a computer-generated randomization list with a ratio of 1:1 and a block size of two."

3b) Important changes to methods after trial commencement (such as eligibility criteria), with reasons

Does your paper address CONSORT subitem 3b? *

Copy and paste relevant sections from the manuscript (include quotes in quotation marks "like this" to indicate direct quotes from your manuscript), or elaborate on this item by providing additional information not in the ms, or briefly explain why the item is not applicable/relevant for your study

No changes occurred.

3b-i) Bug fixes, Downtimes, Content Changes

Bug fixes, Downtimes, Content Changes: ehealth systems are often dynamic systems. A description of changes to methods therefore also includes important changes made on the intervention or comparator during the trial (e.g., major bug fixes or changes in the functionality or content) (5-iii) and other “unexpected events” that may have influenced study design such as staff changes, system failures/downtimes, etc. [2].

	1	2	3	4	5	
subitem not at all important	○	○	○	○	○	essential

Does your paper address subitem 3b-i?

Copy and paste relevant sections from the manuscript (include quotes in quotation marks "like this" to indicate direct quotes from your manuscript), or elaborate on this item by providing additional information not in the ms, or briefly explain why the item is not applicable/relevant for your study

No major bug fixes or changes in the functionality or content occured.

4a) Eligibility criteria for participants

Does your paper address CONSORT subitem 4a? *

Copy and paste relevant sections from the manuscript (include quotes in quotation marks "like this" to indicate direct quotes from your manuscript), or elaborate on this item by providing additional information not in the ms, or briefly explain why the item is not applicable/relevant for your study

"Workers that were 18 years old and older who had access to the internet were invited to participate in the study. Consistent with a universal-prevention approach, elevated insomnia symptoms were not an inclusion criterion. Workers who expressed suicidal ideations — indicated by their response to item 9 from the Beck Depression Inventory II (BDI II, [38] — were excluded. Individuals taking sleep medication were not excluded from the study, but were asked to maintain a constant dose and not change to another sleep medication over the full course of the study."

4a-i) Computer / Internet literacy

Computer / Internet literacy is often an implicit “de facto” eligibility criterion - this should be explicitly clarified.

	1	2	3	4	5	
subitem not at all important	○	○	○	○	○	essential

Does your paper address subitem 4a-i?

Copy and paste relevant sections from the manuscript (include quotes in quotation marks "like this" to indicate direct quotes from your manuscript), or elaborate on this item by providing additional information not in the ms, or briefly explain why the item is not applicable/relevant for your study

"Interested participants registered for the study at www.geton-training.de and had to provide an email address and a first and last name that could be pseudonyms if desired. Once registered, an individual profile on the web-based training platform was created for them."

4a-ii) Open vs. closed, web-based vs. face-to-face assessments:

Open vs. closed, web-based vs. face-to-face assessments: Mention how participants were recruited (online vs. offline), e.g., from an open access website or from a clinic, and clarify if this was a purely web-based trial, or there were face-to-face components (as part of the intervention or for assessment), i.e., to what degree got the study team to know the participant. In online-only trials, clarify if participants were quasi-anonymous and whether having multiple identities was possible or whether technical or logistical measures (e.g., cookies, email confirmation, phone calls) were used to detect/prevent these.

	1	2	3	4	5	
subitem not at all important	○	○	○	○	○	essential

Does your paper address subitem 4a-ii? *

Copy and paste relevant sections from the manuscript (include quotes in quotation marks "like this" to indicate direct quotes from your manuscript), or elaborate on this item by providing additional information not in the ms, or briefly explain why the item is not applicable/relevant for your study

"Recruitment took place via both the media (national telecast and articles) and an e-mail distribution list from the occupational health program provided by one of the largest health insurance companies in Germany."

"Interested participants registered for the study at www.geton-training.de and had to provide an email address and a first and last name that could be pseudonyms if desired. Once registered, an individual profile on the web-based training platform was created for them. Participants received an e-mail with detailed information about the study and a link to the online screening questionnaire on the training platform. All individuals who were employed (1), had access to the Internet (2) and expressed no suicidal ideations (3) were asked to complete the online baseline assessment."

4a-iii) Information giving during recruitment

Information given during recruitment. Specify how participants were briefed for recruitment and in the informed consent procedures (e.g., publish the informed consent documentation as appendix, see also item X26), as this information may have an effect on user self-selection, user expectation and may also bias results.

	1	2	3	4	5	
subitem not at all important	○	○	○	○	○	essential

Does your paper address subitem 4a-iii?

Copy and paste relevant sections from the manuscript (include quotes in quotation marks "like this" to indicate direct quotes from your manuscript), or elaborate on this item by providing additional information not in the ms, or briefly explain why the item is not applicable/relevant for your study

"Once registered, an individual profile on the training platform was created for them. Participants received an e-mail with detailed information about the study and a link to the online screening questionnaire on the web-based training platform. All individuals who were employed (1), had access to the Internet (2) and expressed no suicidal ideations (3) were asked to complete the online baseline assessment and sign the informed consent form, [...]"

4b) Settings and locations where the data were collected

Does your paper address CONSORT subitem 4b? *

Copy and paste relevant sections from the manuscript (include quotes in quotation marks "like this" to indicate direct quotes from your manuscript), or elaborate on this item by providing additional information not in the ms, or briefly explain why the item is not applicable/relevant for your study

"Once registered, an individual profile on the training platform was created for them. Participants received an e-mail with detailed information about the study and a link to the online screening questionnaire on the web-based training platform. All individuals who were employed (1), had access to the Internet (2) and expressed no suicidal ideations (3) were asked to complete the online baseline assessment and sign the informed consent form, [...]"
"Primary and secondary outcomes were self-assessed online at baseline (T1), post treatment (eight weeks post randomization, T2) and at six-month follow-up (four months post treatment, T3; see Figure 1 for details)."

4b-i) Report if outcomes were (self-)assessed through online questionnaires

Clearly report if outcomes were (self-)assessed through online questionnaires (as common in web-based trials) or otherwise.

	1	2	3	4	5	
subitem not at all important	○	○	○	○	○	essential

Does your paper address subitem 4b-i? *

Copy and paste relevant sections from the manuscript (include quotes in quotation marks "like this" to indicate direct quotes from your manuscript), or elaborate on this item by providing additional information not in the ms, or briefly explain why the item is not applicable/relevant for your study

"Primary and secondary outcomes were self-assessed online at baseline (T1), post treatment (eight weeks post randomization, T2) and at six-month follow-up (four months post treatment, T3; see Figure 1 for details)."

4b-ii) Report how institutional affiliations are displayed

Report how institutional affiliations are displayed to potential participants [on ehealth media], as affiliations with prestigious hospitals or universities may affect volunteer rates, use, and reactions with regards to an intervention.(Not a required item – describe only if this may bias results)

	1	2	3	4	5	
subitem not at all important	○	○	○	○	○	essential

Does your paper address subitem 4b-ii?

Copy and paste relevant sections from the manuscript (include quotes in quotation marks "like this" to indicate direct quotes from your manuscript), or elaborate on this item by providing additional information not in the ms, or briefly explain why the item is not applicable/relevant for your study

"Recruitment took place via both the media (national telecast and articles) and an e-mail distribution list from the occupational health program provided by one of the largest health insurance companies in Germany."

5) The interventions for each group with sufficient details to allow replication, including how and when they were actually administered

5-i) Mention names, credential, affiliations of the developers, sponsors, and owners

Mention names, credential, affiliations of the developers, sponsors, and owners [6] (if authors/evaluators are owners or developer of the software, this needs to be declared in a “Conflict of interest” section or mentioned elsewhere in the manuscript).

	1	2	3	4	5	
subitem not at all important	○	○	○	○	○	essential

Does your paper address subitem 5-i?

Copy and paste relevant sections from the manuscript (include quotes in quotation marks "like this" to indicate direct quotes from your manuscript), or elaborate on this item by providing additional information not in the ms, or briefly explain why the item is not applicable/relevant for your study

The intervention was developed at the Leuphana University Lueneburg, Germany by part of the authors of this article using a web-based platform (https://get-on.minddistrict.de). The European Union funded this study, project number: EFRE: CCI 2007DE161PR001.

5-ii) Describe the history/development process

Describe the history/development process of the application and previous formative evaluations (e.g., focus groups, usability testing), as these will have an impact on adoption/use rates and help with interpreting results.

	1	2	3	4	5	
subitem not at all important	○	○	○	○	○	essential

Does your paper address subitem 5-ii?

Copy and paste relevant sections from the manuscript (include quotes in quotation marks "like this" to indicate direct quotes from your manuscript), or elaborate on this item by providing additional information not in the ms, or briefly explain why the item is not applicable/relevant for your study

"In two previous studies, GET.ON Recovery, the training used for the current study, was evaluated in school teachers [34,35]."

5-iii) Revisions and updating

Revisions and updating. Clearly mention the date and/or version number of the application/intervention (and comparator, if applicable) evaluated, or describe whether the intervention underwent major changes during the evaluation process, or whether the development and/or content was “frozen” during the trial. Describe dynamic components such as news feeds or changing content which may have an impact on the replicability of the intervention (for unexpected events see item 3b).

	1	2	3	4	5	
subitem not at all important	○	○	○	○	○	essential

Does your paper address subitem 5-iii?

Copy and paste relevant sections from the manuscript (include quotes in quotation marks "like this" to indicate direct quotes from your manuscript), or elaborate on this item by providing additional information not in the ms, or briefly explain why the item is not applicable/relevant for your study

The intervention content was "frozen" during the trial.

5-iv) Quality assurance methods

Provide information on quality assurance methods to ensure accuracy and quality of information provided [1], if applicable.

	1	2	3	4	5	
subitem not at all important	○	○	○	○	○	essential

Does your paper address subitem 5-iv?

Copy and paste relevant sections from the manuscript (include quotes in quotation marks "like this" to indicate direct quotes from your manuscript), or elaborate on this item by providing additional information not in the ms, or briefly explain why the item is not applicable/relevant for your study

Meine Antwort

5-v) Ensure replicability by publishing the source code, and/or providing screenshots/screen-capture video, and/or providing flowcharts of the algorithms used

Ensure replicability by publishing the source code, and/or providing screenshots/screen-capture video, and/or providing flowcharts of the algorithms used. Replicability (i.e., other researchers should in principle be able to replicate the study) is a hallmark of scientific reporting.

	1	2	3	4	5	
subitem not at all important	○	○	○	○	○	essential

Does your paper address subitem 5-v?

Copy and paste relevant sections from the manuscript (include quotes in quotation marks "like this" to indicate direct quotes from your manuscript), or elaborate on this item by providing additional information not in the ms, or briefly explain why the item is not applicable/relevant for your study

Screenshots of the intervention are included in the supplemental material.

5-vi) Digital preservation

Digital preservation: Provide the URL of the application, but as the intervention is likely to change or disappear over the course of the years; also make sure the intervention is archived (Internet Archive, webcitation.org, and/or publishing the source code or screenshots/videos alongside the article). As pages behind login screens cannot be archived, consider creating demo pages which are accessible without login.

	1	2	3	4	5	
subitem not at all important	○	○	○	○	○	essential

Does your paper address subitem 5-vi?

Copy and paste relevant sections from the manuscript (include quotes in quotation marks "like this" to indicate direct quotes from your manuscript), or elaborate on this item by providing additional information not in the ms, or briefly explain why the item is not applicable/relevant for your study

The log-in page of the web-based platform can be found here:
http://www.webcitation.org/75HEcVike
Screenshots of the intervention are included in the supplemental material.

5-vii) Access

Access: Describe how participants accessed the application, in what setting/context, if they had to pay (or were paid) or not, whether they had to be a member of specific group. If known, describe how participants obtained "access to the platform and Internet" [1]. To ensure access for editors/reviewers/readers, consider to provide a "backdoor" login account or demo mode for reviewers/readers to explore the application (also important for archiving purposes, see vi).

	1	2	3	4	5	
subitem not at all important	○	○	○	○	○	essential

Does your paper address subitem 5-vii? *

Copy and paste relevant sections from the manuscript (include quotes in quotation marks "like this" to indicate direct quotes from your manuscript), or elaborate on this item by providing additional information not in the ms, or briefly explain why the item is not applicable/relevant for your study

"Interested participants registered for the study at www.geton-training.de and had to provide an email address and a first and last name that could be pseudonyms if desired. Once registered, an individual profile on the web-based training platform was created for them."

5-viii) Mode of delivery, features/functionalities/components of the intervention and comparator, and the theoretical framework

Describe mode of delivery, features/functionalities/components of the intervention and comparator, and the theoretical framework [6] used to design them (instructional strategy [1], behaviour change techniques, persuasive features, etc., see e.g., [7, 8] for terminology). This includes an in-depth description of the content (including where it is coming from and who developed it) [1]," whether [and how] it is tailored to individual circumstances and allows users to track their progress and receive feedback" [6]. This also includes a description of communication delivery channels and – if computer-mediated communication is a component – whether communication was synchronous or asynchronous [6]. It also includes information on presentation strategies [1], including page design principles, average amount of text on pages, presence of hyperlinks to other resources, etc. [1].

	1	2	3	4	5	
subitem not at all important	○	○	○	○	○	essential

Does your paper address subitem 5-viii? *

Copy and paste relevant sections from the manuscript (include quotes in quotation marks "like this" to indicate direct quotes from your manuscript), or elaborate on this item by providing additional information not in the ms, or briefly explain why the item is not applicable/relevant for your study

"The present GET.ON Recovery training is a self-help, work-life-domain-tailored web-based training grounded in the principles of CBT-I. The training consists of six one-week modules, each lasting 45-60 minutes. The intervention was developed for workers who have recurring problems initiating and/or maintaining sleep, tend to ruminate about their work, and have problems detaching from work. Therefore, the training not only includes well-established CBT-I methods [39], but also various techniques to reduce work-related cognitive activation in the evening. The modules focus on the following subjects: In Module 1, subjects are provided psychoeducation on healthy sleep; plan which sleep hygiene rules they are going to follow over the subsequent week; and are introduced to the concept of a sleep diary. Module 2 focuses on sleep restriction and stimulus control. In Module 3, boundary tactics to support mental detachment from work are targeted; and a gratitude diary is introduced to prevent ruminative thoughts prior to sleeping. During module 4, participants receive information on work-related rumination and worry, including their impact on sleep, and techniques (e.g., relaxation exercises) to overcome them. Module 5 teaches subjects how to employ MCT to overcome ruminating thoughts. Finally, in Module 6, participants reflect on strategies they tried during their training and their potential future application. Every session includes subjects planning to insert recreational activities into their daily life, as per the behavioural activation approach. Additionally, at the end of each session, participants are asked to select at least one exercise to do over the following week. The training included interactive exercises, audio/video files, and downloadable material and was presented on a secured Web-based platform. Since training was self-help, participants received nothing but technical support via email."

"Participants, which were allocated to the training group had immediate access to the intervention. Controls received access to the training after their six-month follow-up assessment; in the meantime, they had full access to the usual care offered by routine healthcare services throughout the trial."

5-ix) Describe use parameters

Describe use parameters (e.g., intended "doses" and optimal timing for use). Clarify what instructions or recommendations were given to the user, e.g., regarding timing, frequency, heaviness of use, if any, or was the intervention used ad libitum.

	1	2	3	4	5	
subitem not at all important	○	○	○	○	○	essential

Does your paper address subitem 5-ix?

Copy and paste relevant sections from the manuscript (include quotes in quotation marks "like this" to indicate direct quotes from your manuscript), or elaborate on this item by providing additional information not in the ms, or briefly explain why the item is not applicable/relevant for your study

"The training consists of six one-week modules, each lasting 45-60 minutes."

5-x) Clarify the level of human involvement

Clarify the level of human involvement (care providers or health professionals, also technical assistance) in the e-intervention or as co-intervention (detail number and expertise of professionals involved, if any, as well as "type of assistance offered, the timing and frequency of the support, how it is initiated, and the medium by which the assistance is delivered". It may be necessary to distinguish between the level of human involvement required for the trial, and the level of human involvement required for a routine application outside of a RCT setting (discuss under item 21 – generalizability).

	1	2	3	4	5	
subitem not at all important	○	○	○	○	○	essential

Does your paper address subitem 5-x?

Copy and paste relevant sections from the manuscript (include quotes in quotation marks "like this" to indicate direct quotes from your manuscript), or elaborate on this item by providing additional information not in the ms, or briefly explain why the item is not applicable/relevant for your study

"Since training was self-help, participants received nothing but technical support via email."

5-xi) Report any prompts/reminders used

Report any prompts/reminders used: Clarify if there were prompts (letters, emails, phone calls, SMS) to use the application, what triggered them, frequency etc. It may be necessary to distinguish between the level of prompts/reminders required for the trial, and the level of prompts/reminders for a routine application outside of a RCT setting (discuss under item 21 – generalizability).

	1	2	3	4	5	
subitem not at all important	○	○	○	○	○	essential

Does your paper address subitem 5-xi? *

Copy and paste relevant sections from the manuscript (include quotes in quotation marks "like this" to indicate direct quotes from your manuscript), or elaborate on this item by providing additional information not in the ms, or briefly explain why the item is not applicable/relevant for your study

"Since training was self-help, participants received nothing but technical support via email."

5-xii) Describe any co-interventions (incl. training/support)

Describe any co-interventions (incl. training/support): Clearly state any interventions that are provided in addition to the targeted eHealth intervention, as ehealth intervention may not be designed as stand-alone intervention. This includes training sessions and support [1]. It may be necessary to distinguish between the level of training required for the trial, and the level of training for a routine application outside of a RCT setting (discuss under item 21 – generalizability.

	1	2	3	4	5	
subitem not at all important	○	○	○	○	○	essential

Does your paper address subitem 5-xii? *

Copy and paste relevant sections from the manuscript (include quotes in quotation marks "like this" to indicate direct quotes from your manuscript), or elaborate on this item by providing additional information not in the ms, or briefly explain why the item is not applicable/relevant for your study

"In this randomized trial, subjects were randomly assigned to either receive access to the iCBT-I (GET.ON Recovery) or receive usual care (as controls)."

6a) Completely defined pre-specified primary and secondary outcome measures, including how and when they were assessed

Does your paper address CONSORT subitem 6a? *

Copy and paste relevant sections from the manuscript (include quotes in quotation marks "like this" to indicate direct quotes from your manuscript), or elaborate on this item by providing additional information not in the ms, or briefly explain why the item is not applicable/relevant for your study

"In this randomized trial, subjects were randomly assigned to either receive access to the iCBT-I (GET.ON Recovery) or receive usual care (as controls). Primary and secondary outcomes were self-assessed online at baseline (T1), post treatment (eight weeks post randomization, T2) and at six-month follow-up (four months post treatment, T3; see Figure 1 for details)."
insomnia severity index [ISI]; Center for Epidemiological Studies' Depression Scale [CES-D]; recuperation in sleep subscale [SF-B]; Recreation Experience and Activity Questionnaire [ReaQ]; work ability (single-item score from the Work Ability Index [WAI]; absenteeism and presenteeism over the past three months, German Version of the Trimbos/iMTA questionnaire for costs associated with psychiatric illness (TiC-P-G); Cognitive Irritation subscale [IS]; Penn State Worry Questionnaire, Ultra Brief Version, past week, [PSWQ-PW]; Client Satisfaction Questionnaire [CSQ-8]

6a-i) Online questionnaires: describe if they were validated for online use and apply CHERRIES items to describe how the questionnaires were designed/deployed

If outcomes were obtained through online questionnaires, describe if they were validated for online use and apply CHERRIES items to describe how the questionnaires were designed/deployed [9].

	1	2	3	4	5	
subitem not at all important	○	○	○	○	○	essential

Does your paper address subitem 6a-i?

Copy and paste relevant sections from manuscript text

Meine Antwort

6a-ii) Describe whether and how "use" (including intensity of use/dosage) was defined/measured/monitored

Describe whether and how "use" (including intensity of use/dosage) was defined/measured/monitored (logins, logfile analysis, etc.). Use/adoption metrics are important process outcomes that should be reported in any ehealth trial.

	1	2	3	4	5	
subitem not at all important	○	○	○	○	○	essential

Does your paper address subitem 6a-ii?

Copy and paste relevant sections from manuscript text

The number of completed sessions was used as a measure of adherence. The results are reported in the article.

6a-iii) Describe whether, how, and when qualitative feedback from participants was obtained

Describe whether, how, and when qualitative feedback from participants was obtained (e.g., through emails, feedback forms, interviews, focus groups).

	1	2	3	4	5	
subitem not at all important	○	○	○	○	○	essential

Does your paper address subitem 6a-iii?

Copy and paste relevant sections from manuscript text

Client satisfaction was obtained at post-test and is reported in the result

6b) Any changes to trial outcomes after the trial commenced, with reasons

Does your paper address CONSORT subitem 6b? *

Copy and paste relevant sections from the manuscript (include quotes in quotation marks "like this" to indicate direct quotes from your manuscript), or elaborate on this item by providing additional information not in the ms, or briefly explain why the item is not applicable/relevant for your study

There were no changes to trial aoutcomes.

7a) How sample size was determined

NPT: When applicable, details of whether and how the clustering by care provides or centers was addressed

7a-i) Describe whether and how expected attrition was taken into account when calculating the sample size

Describe whether and how expected attrition was taken into account when calculating the sample size.

	1	2	3	4	5	
subitem not at all important	○	○	○	○	○	essential

Does your paper address subitem 7a-i?

Copy and paste relevant sections from manuscript title (include quotes in quotation marks "like this" to indicate direct quotes from your manuscript), or elaborate on this item by providing additional information not in the ms, or briefly explain why the item is not applicable/relevant for your study

"Previous studies on this training have yielded treatment effects for insomnia severity ranging from d = 1.37 to d = 1.45 in teachers with elevated insomnia symptoms. However, studies following a universal health prevention approach are usually associated with smaller effect sizes [36,37]. Thus, our sample size calculation assumed an effect size of d = 0.4 for the primary outcome (insomnia severity), due to our inclusion of less-symptomatic workers. We, therefore, estimated needing 200 participants to achieve 80% power (β = .80) and 95% confidence (α = .05) in two-tailed analysis."

7b) When applicable, explanation of any interim analyses and stopping guidelines

Does your paper address CONSORT subitem 7b? *

Copy and paste relevant sections from the manuscript (include quotes in quotation marks "like this" to indicate direct quotes from your manuscript), or elaborate on this item by providing additional information not in the ms, or briefly explain why the item is not applicable/relevant for your study

Not applicable

8a) Method used to generate the random allocation sequence

NPT: When applicable, how care providers were allocated to each trial group

Does your paper address CONSORT subitem 8a? *

Copy and paste relevant sections from the manuscript (include quotes in quotation marks "like this" to indicate direct quotes from your manuscript), or elaborate on this item by providing additional information not in the ms, or briefly explain why the item is not applicable/relevant for your study

"[...] after which they were randomized to one of the two treatment arms, using a computer-generated randomization list with a ratio of 1:1 and a block size of two. The randomization list was generated and randomization performed by two researchers in our department who were not otherwise involved in the current study."

8b) Type of randomisation; details of any restriction (such as blocking and block size)

Does your paper address CONSORT subitem 8b? *

Copy and paste relevant sections from the manuscript (include quotes in quotation marks "like this" to indicate direct quotes from your manuscript), or elaborate on this item by providing additional information not in the ms, or briefly explain why the item is not applicable/relevant for your study

"[...] after which they were randomized to one of the two treatment arms, using a computer-generated randomization list with a ratio of 1:1 and a block size of two. The randomization list was generated and randomization performed by two researchers in our department who were not otherwise involved in the current study."

9) Mechanism used to implement the random allocation sequence (such as sequentially numbered containers), describing any steps taken to conceal the sequence until interventions were assigned

Does your paper address CONSORT subitem 9? *

Copy and paste relevant sections from the manuscript (include quotes in quotation marks "like this" to indicate direct quotes from your manuscript), or elaborate on this item by providing additional information not in the ms, or briefly explain why the item is not applicable/relevant for your study

"[...] after which they were randomized to one of the two treatment arms, using a computer-generated randomization list with a ratio of 1:1 and a block size of two. The randomization list was generated and randomization performed by two researchers in our department who were not otherwise involved in the current study."

10) Who generated the random allocation sequence, who enrolled participants, and who assigned participants to interventions

Does your paper address CONSORT subitem 10? *

Copy and paste relevant sections from the manuscript (include quotes in quotation marks "like this" to indicate direct quotes from your manuscript), or elaborate on this item by providing additional information not in the ms, or briefly explain why the item is not applicable/relevant for your study

"The randomization list was generated and randomization performed by two researchers in our department who were not otherwise involved in the current study."

11a) If done, who was blinded after assignment to interventions (for example, participants, care providers, those assessing outcomes) and how

NPT: Whether or not administering co-interventions were blinded to group assignment

11a-i) Specify who was blinded, and who wasn't

Specify who was blinded, and who wasn't. Usually, in web-based trials it is not possible to blind the participants [1, 3] (this should be clearly acknowledged), but it may be possible to blind outcome assessors, those doing data analysis or those administering co-interventions (if any).

	1	2	3	4	5	
subitem not at all important	○	○	○	○	○	essential

Does your paper address subitem 11a-i? *

Copy and paste relevant sections from the manuscript (include quotes in quotation marks "like this" to indicate direct quotes from your manuscript), or elaborate on this item by providing additional information not in the ms, or briefly explain why the item is not applicable/relevant for your study

"Blinding to group allocation was not feasible. The participants were informed about the randomization outcome via e-mail."

11a-ii) Discuss e.g., whether participants knew which intervention was the “intervention of interest” and which one was the “comparator”

Informed consent procedures (4a-ii) can create biases and certain expectations - discuss e.g., whether participants knew which intervention was the “intervention of interest” and which one was the “comparator”.

	1	2	3	4	5	
subitem not at all important	○	○	○	○	○	essential

Does your paper address subitem 11a-ii?

Copy and paste relevant sections from the manuscript (include quotes in quotation marks "like this" to indicate direct quotes from your manuscript), or elaborate on this item by providing additional information not in the ms, or briefly explain why the item is not applicable/relevant for your study

"The participants were informed about the randomization outcome via e-mail."

11b) If relevant, description of the similarity of interventions

(this item is usually not relevant for ehealth trials as it refers to similarity of a placebo or sham intervention to a active medication/intervention)

Does your paper address CONSORT subitem 11b? *

Copy and paste relevant sections from the manuscript (include quotes in quotation marks "like this" to indicate direct quotes from your manuscript), or elaborate on this item by providing additional information not in the ms, or briefly explain why the item is not applicable/relevant for your study

Not applicable

12a) Statistical methods used to compare groups for primary and secondary outcomes

NPT: When applicable, details of whether and how the clustering by care providers or centers was addressed

Does your paper address CONSORT subitem 12a? *

Copy and paste relevant sections from the manuscript (include quotes in quotation marks "like this" to indicate direct quotes from your manuscript), or elaborate on this item by providing additional information not in the ms, or briefly explain why the item is not applicable/relevant for your study

"All analyses are reported in compliance with the CONSORT-EHEALTH guidelines for improving and standardizing the report of Web-based and mobile health interventions [53] using intention-to-treat (ITT) procedures. Analyses were performed using IBM SPSS v. 24. Reported p-values are two-sided, with the a priori threshold for statistical significance set at 0.05."
"The intervention's efficacy was compared to being in the control group at both, T2 and T3, using ITT analysis of covariance (ANCOVA), with baseline scores for the respective primary and secondary outcomes as covariates. Cohen´s ds and corresponding 95% confidence intervals (CI) were computed for T2 and T3, comparing the means (M) and standard deviations (SD) for both the iCBT-I and control groups immediately post treatment and at six-month follow-up."

12a-i) Imputation techniques to deal with attrition / missing values

Imputation techniques to deal with attrition / missing values: Not all participants will use the intervention/comparator as intended and attrition is typically high in ehealth trials. Specify how participants who did not use the application or dropped out from the trial were treated in the statistical analysis (a complete case analysis is strongly discouraged, and simple imputation techniques such as LOCF may also be problematic [4]).

	1	2	3	4	5	
subitem not at all important	○	○	○	○	○	essential

Does your paper address subitem 12a-i? *

Copy and paste relevant sections from the manuscript (include quotes in quotation marks "like this" to indicate direct quotes from your manuscript), or elaborate on this item by providing additional information not in the ms, or briefly explain why the item is not applicable/relevant for your study

"Little's Missing Completely at Random-Test indicated that the data missing for the observed outcomes occurred completely at random ($\chi 2$ = 126.21, P = 0.11). Therefore, following intention-to-treat (ITT) principles, multiple imputations with 10 estimates per missing value were conducted to handle missing data."

12b) Methods for additional analyses, such as subgroup analyses and adjusted analyses

Does your paper address CONSORT subitem 12b? *

Copy and paste relevant sections from the manuscript (include quotes in quotation marks "like this" to indicate direct quotes from your manuscript), or elaborate on this item by providing additional information not in the ms, or briefly explain why the item is not applicable/relevant for your study

"Reliable change and symptom-free status
To detect reliable changes in insomnia severity from T1 to T2, and from T1 to T3, a reliable change index (RCI) was calculated [54]. Based upon this, ISI score differences of more than 5.01 points — between T1 and either T2 or T3 — were considered a reliable change in an individual subject [34,35]. To detect potential negative effects of the intervention, the number of participants with reliable symptom deterioration, according to the RCI, was assessed. To assess symptom-free status an ISI score below 8 was considered symptom free [41]. Additionally, the number needed to treat (NNT) was calculated by comparing the two groups for the number of participants (1) with or without a reliable change in insomnia severity, and (2) who became versus failed to become symptom free.
Sensitivity Analyses
To test the robustness of the results, both complete-case analyses and moderator analyses were conducted. Complete-case analyses were performed, only including participants with a complete data set at both T1 and T2.
The study sample was heterogeneous with respect to insomnia severity at baseline, as there were no inclusion criteria regarding the severity of the latter. To assess whether the intervention´s effect on insomnia severity at post treatment and at six-month follow-up is moderated by different levels of insomnia severity at baseline (prior to receiving the iCBT-I), simple moderation analysis was conducted using the PROCESS software for SPSS (model 1). For significant moderation, the Johnson-Neyman (J-N) procedure was employed [55] to identify the specific values of the moderator at which the groups differed significantly on the primary outcome at both assessment points.
Mediators of interventional effects
To examine both forms of cognitive activation, measured as worry and work-related rumination, as potential mediators of treatment effects on the primary outcome at T2, parallel multiple mediation analysis was conducted using the PROCESS software for SPSS (model 4), with bias-corrected bootstraps based upon 10.000 bootstrap samples [55]. To establish temporal precedence, the post-intervention scores of the mediators and six-month follow-up scores for the primary outcome were used. Following the recommendation of Valente and McKinnon [56], baseline scores for the mediating variables and primary outcome were included as covariates in the model. Statistical significance of the mediation was achieved if the estimated 95% CI for the indirect effect did not overlap zero [55]."

X26) REB/IRB Approval and Ethical Considerations [recommended as subheading under "Methods"] (not a CONSORT item)

X26-i) Comment on ethics committee approval

	1	2	3	4	5	
subitem not at all important	○	○	○	○	○	essential

Does your paper address subitem X26-i?

Copy and paste relevant sections from the manuscript (include quotes in quotation marks "like this" to indicate direct quotes from your manuscript), or elaborate on this item by providing additional information not in the ms, or briefly explain why the item is not applicable/relevant for your study

"The study was approved by the Ethics Committee of Leuphana University of Lueneburg (Lehr201411_Schlaftraining) and registered as DRKS00007142 in the German Clinical Trials Registry (DRKS)."

x26-ii) Outline informed consent procedures

Outline informed consent procedures e.g., if consent was obtained offline or online (how? Checkbox, etc.?), and what information was provided (see 4a-ii). See [6] for some items to be included in informed consent documents.

	1	2	3	4	5	
subitem not at all important	○	○	○	○	○	essential

Does your paper address subitem X26-ii?

Copy and paste relevant sections from the manuscript (include quotes in quotation marks "like this" to indicate direct quotes from your manuscript), or elaborate on this item by providing additional information not in the ms, or briefly explain why the item is not applicable/relevant for your study

"Participants received an e-mail with detailed information about the study and a link to the online screening questionnaire on the training platform. All individuals who were employed (1), had access to the Internet (2) and expressed no suicidal ideations (3) were asked to complete the online baseline assessment and sign the informed consent form, [...]"

X26-iii) Safety and security procedures

Safety and security procedures, incl. privacy considerations, and any steps taken to reduce the likelihood or detection of harm (e.g., education and training, availability of a hotline)

	1	2	3	4	5	
subitem not at all important	○	○	○	○	○	essential

Does your paper address subitem X26-iii?

Copy and paste relevant sections from the manuscript (include quotes in quotation marks "like this" to indicate direct quotes from your manuscript), or elaborate on this item by providing additional information not in the ms, or briefly explain why the item is not applicable/relevant for your study

"Interested participants registered for the study at www.geton-training.de and had to provide an email address and a first and last name that could be pseudonyms if desired."
"Workers who expressed suicidal ideations — indicated by their response to item 9 from the Beck Depression Inventory II (BDI II, [38] — were excluded."

RESULTS

13a) For each group, the numbers of participants who were randomly assigned, received intended treatment, and were analysed for the primary outcome

NPT: The number of care providers or centers performing the intervention in each group and the number of patients treated by each care provider in each center

Does your paper address CONSORT subitem 13a? *

Copy and paste relevant sections from the manuscript (include quotes in quotation marks "like this" to indicate direct quotes from your manuscript), or elaborate on this item by providing additional information not in the ms, or briefly explain why the item is not applicable/relevant for your study

Please refer to CONSORT Flow diagram. An intention-to-treat analysis was conducted.

"In total, 177 individuals were randomized to either receive the iCBT-I (n = 88) or be controls (n = 89). Since the 10-month funding period that we were granted was inadequate to recruit the originally-intended sample of 200 subjects, and further recruitment was impossible, the final sample size was 177. With 177 subjects, the trial had 80% power to detect an intervention effect of d = 0.42."

"All participants completed the baseline assessment. At eight weeks (T2), 25% (n = 33 in the iCBT-I group and n = 11 controls) of the data were missing; at T3, 28% (n = 42 and n = 8, respectively)."

"Of the 88 individuals who were assigned to the iCBT-I group, 17 (19%) dropped out before completing Module 1 of the intervention. Module 1 was completed by 71 (81%), Module 2 by 56 (64%), Module 3 by 51 (58%), Module 4 by 46 (52%), Module 5 by 39 (44%), and all Modules by 35 (40%) participants."

13b) For each group, losses and exclusions after randomisation, together with reasons

Does your paper address CONSORT subitem 13b? (NOTE: Preferably, this is shown in a CONSORT flow diagram) *

Copy and paste relevant sections from the manuscript (include quotes in quotation marks "like this" to indicate direct quotes from your manuscript), or elaborate on this item by providing additional information not in the ms, or briefly explain why the item is not applicable/relevant for your study

Please refer to CONSORT Flow diagram

13b-i) Attrition diagram

Strongly recommended: An attrition diagram (e.g., proportion of participants still logging in or using the intervention/comparator in each group plotted over time, similar to a survival curve) or other figures or tables demonstrating usage/dose/engagement.

	1	2	3	4	5	
subitem not at all important	○	○	○	○	○	essential

Does your paper address subitem 13b-i?

Copy and paste relevant sections from the manuscript or cite the figure number if applicable (include quotes in quotation marks "like this" to indicate direct quotes from your manuscript), or elaborate on this item by providing additional information not in the ms, or briefly explain why the item is not applicable/relevant for your study

"Of the 88 individuals who were assigned to the iCBT-I group, 17 (19%) dropped out before completing Module 1 of the intervention. Module 1 was completed by 71 (81%), Module 2 by 56 (64%), Module 3 by 51 (58%), Module 4 by 46 (52%), Module 5 by 39 (44%), and all Modules by 35 (40%) participants."

14a) Dates defining the periods of recruitment and follow-up

Does your paper address CONSORT subitem 14a? *

Copy and paste relevant sections from the manuscript (include quotes in quotation marks "like this" to indicate direct quotes from your manuscript), or elaborate on this item by providing additional information not in the ms, or briefly explain why the item is not applicable/relevant for your study

"Primary and secondary outcomes were self-assessed online at baseline (T1), post treatment (eight weeks post randomization, T2) and at six-month follow-up (four months post treatment, T3; see Figure 1 for details)."

14a-i) Indicate if critical "secular events" fell into the study period

Indicate if critical "secular events" fell into the study period, e.g., significant changes in Internet resources available or "changes in computer hardware or Internet delivery resources"

	1	2	3	4	5	
subitem not at all important	○	○	○	○	○	essential

Does your paper address subitem 14a-i?

Copy and paste relevant sections from the manuscript (include quotes in quotation marks "like this" to indicate direct quotes from your manuscript), or elaborate on this item by providing additional information not in the ms, or briefly explain why the item is not applicable/relevant for your study

No secular events occurred.

14b) Why the trial ended or was stopped (early)

Does your paper address CONSORT subitem 14b? *

Copy and paste relevant sections from the manuscript (include quotes in quotation marks "like this" to indicate direct quotes from your manuscript), or elaborate on this item by providing additional information not in the ms, or briefly explain why the item is not applicable/relevant for your study

"In total, 177 individuals were randomized to either receive the iCBT-I (n = 88) or be controls (n = 89). Since the 10-month funding period that we were granted was inadequate to recruit the originally-intended sample of 200 subjects, and further recruitment was impossible, the final sample size was 177. With 177 subjects, the trial had 80% power to detect an intervention effect of d = 0.42."

15) A table showing baseline demographic and clinical characteristics for each group

NPT: When applicable, a description of care providers (case volume, qualification, expertise, etc.) and centers (volume) in each group

Does your paper address CONSORT subitem 15? *

Copy and paste relevant sections from the manuscript (include quotes in quotation marks "like this" to indicate direct quotes from your manuscript), or elaborate on this item by providing additional information not in the ms, or briefly explain why the item is not applicable/relevant for your study

Please refer to table 1 (baseline characteristics) and table 2 (means and standard deviations for each group)

15-i) Report demographics associated with digital divide issues

In ehealth trials it is particularly important to report demographics associated with digital divide issues, such as age, education, gender, social-economic status, computer/Internet/ehealth literacy of the participants, if known.

	1	2	3	4	5	
subitem not at all important	○	○	○	○	○	essential

Does your paper address subitem 15-i? *

Copy and paste relevant sections from the manuscript (include quotes in quotation marks "like this" to indicate direct quotes from your manuscript), or elaborate on this item by providing additional information not in the ms, or briefly explain why the item is not applicable/relevant for your study

Please refer to table 1 (baseline characteristics)

16) For each group, number of participants (denominator) included in each analysis and whether the analysis was by original assigned groups

16-i) Report multiple "denominators" and provide definitions

Report multiple "denominators" and provide definitions: Report N's (and effect sizes) "across a range of study participation [and use] thresholds" [1], e.g., N exposed, N consented, N used more than x times, N used more than y weeks, N participants "used" the intervention/comparator at specific pre-defined time points of interest (in absolute and relative numbers per group). Always clearly define "use" of the intervention.

	1	2	3	4	5	
subitem not at all important	○	○	○	○	○	essential

Does your paper address subitem 16-i? *

Copy and paste relevant sections from the manuscript (include quotes in quotation marks "like this" to indicate direct quotes from your manuscript), or elaborate on this item by providing additional information not in the ms, or briefly explain why the item is not applicable/relevant for your study

Please refer to the results section

16-ii) Primary analysis should be intent-to-treat

Primary analysis should be intent-to-treat, secondary analyses could include comparing only “users”, with the appropriate caveats that this is no longer a randomized sample (see 18-i).

	1	2	3	4	5	
subitem not at all important	○	○	○	○	○	essential

Does your paper address subitem 16-ii?

Copy and paste relevant sections from the manuscript (include quotes in quotation marks "like this" to indicate direct quotes from your manuscript), or elaborate on this item by providing additional information not in the ms, or briefly explain why the item is not applicable/relevant for your study

Intention-to-treat analyses have been performed.

17a) For each primary and secondary outcome, results for each group, and the estimated effect size and its precision (such as 95% confidence interval)

Does your paper address CONSORT subitem 17a? *

Copy and paste relevant sections from the manuscript (include quotes in quotation marks "like this" to indicate direct quotes from your manuscript), or elaborate on this item by providing additional information not in the ms, or briefly explain why the item is not applicable/relevant for your study

Please refer to table 3 (Results of the ANCOVAs and Cohen´s d for the primary and secondary outcome measures)

17a-i) Presentation of process outcomes such as metrics of use and intensity of use

In addition to primary/secondary (clinical) outcomes, the presentation of process outcomes such as metrics of use and intensity of use (dose, exposure) and their operational definitions is critical. This does not only refer to metrics of attrition (13-b) (often a binary variable), but also to more continuous exposure metrics such as “average session length”. These must be accompanied by a technical description how a metric like a “session” is defined (e.g., timeout after idle time) [1] (report under item 6a).

	1	2	3	4	5	
subitem not at all important	○	○	○	○	○	essential

Does your paper address subitem 17a-i?

Copy and paste relevant sections from the manuscript (include quotes in quotation marks "like this" to indicate direct quotes from your manuscript), or elaborate on this item by providing additional information not in the ms, or briefly explain why the item is not applicable/relevant for your study

"Of the 88 individuals who were assigned to the iCBT-I group, 17 (19%) dropped out before completing Module 1 of the intervention. Module 1 was completed by 71 (81%), Module 2 by 56 (64%), Module 3 by 51 (58%), Module 4 by 46 (52%), Module 5 by 39 (44%), and all Modules by 35 (40%) participants. On average, 3.4 modules (SD = 2.3) were completed in the training group. Only three participants reported their reason for dropout. Stated reasons were “technical problems”, “lack of time” and “already sufficient help before the last module”."

17b) For binary outcomes, presentation of both absolute and relative effect sizes is recommended

Does your paper address CONSORT subitem 17b? *

Copy and paste relevant sections from the manuscript (include quotes in quotation marks "like this" to indicate direct quotes from your manuscript), or elaborate on this item by providing additional information not in the ms, or briefly explain why the item is not applicable/relevant for your study

"Reliable change and symptom-free status
At T2, more participants in the iCBT-I group (n = 50, 57%) reported a reliable improvement in insomnia severity than controls (n = 15, 17%), which yielded a NNT of 2.50 (95% CI 1.89 — 3.70) to achieve reliable improvement from baseline to post treatment. Also at T3, more participants in the iCBT-I group reported a reliable improvement in insomnia severity [N = 51 (58%)] than in the control group (n = 18, 20%), corresponding to a NNT of 2.65 (95% CI 1.96 — 4.09).
At T2, no iCBT-I group member versus one control subject experienced reliable deterioration (T2 score of > 5.01 above the T1 score). At six-month follow-up, corresponding numbers were one and two.
Two subjects who received the intervention and three controls were symptom-free at baseline and excluded from further symptom-free analysis. Significantly more iCBT-I group participants (n = 22, 26%) were symptom-free at T2 than controls (n = 6, 7%), corresponding to a NNT of 5.38 (95% CI 3.41 — 12.62). The same was true at T3, with significantly more iCBT-I group participants than controls claiming to be symptom free (n = 14, 16%) versus n = 4 (5%), generating a NNT of 8.60 (95% CI 4.85 — 37.80)."

18) Results of any other analyses performed, including subgroup analyses and adjusted analyses, distinguishing pre-specified from exploratory

Does your paper address CONSORT subitem 18? *

Copy and paste relevant sections from the manuscript (include quotes in quotation marks "like this" to indicate direct quotes from your manuscript), or elaborate on this item by providing additional information not in the ms, or briefly explain why the item is not applicable/relevant for your study

"Sensitivity Analyses
Complete-case analysis supported the results of the ITT analysis, revealing even larger effects for the primary outcome at both T2 ($F_{1, 130}$ = 52.04, P < 0.001; Cohen´s d = 1.24, 95% CI 0.58 – 1.30) and T3 ($F_{1, 123}$ = 36.89, P < 0.001; Cohen´s d = 0.95, 95% CI 0.57 – 1.33). Moderation analysis showed a significant group x baseline insomnia severity interaction (b = -0.55, SE = 0.13, t = -4.30, P = 0.000, 95% CI -0.80 — -0.30) immediately post treatment, indicating that the intervention´s effect on insomnia severity was moderated by the severity of insomnia at baseline. Probing this effect, using the J-N technique, revealed a baseline ISI score of 11.20 as the point of transition between a statistically-nonsignificant and significant effect of iCBT-I on post treatment insomnia severity (b = -1.67, SE =0.84, t = -1.97, P = 0.05, 95% CI -3.33 — 0.00). This means that, at T2, a significant group difference was identified for participants scoring ≥ 11 on the baseline insomnia severity score (88.1% of all participants). No significant moderation effect of baseline levels of insomnia severity was detected for the intervention´s effect on insomnia severity at the six-month follow-up assessment (b = -0.21, SE = 0.12, t = -1.68, P = 0.10, 95% CI -0.45 — 0.04)."

"Mediators of interventional effects
As depicted in Figure 2, the results of parallel multiple mediation analysis indicated that both forms of cognitive activation — work-related rumination (indirect effect: b = -0.99, SE =.35, 95% Boot CI -1.80 – -0.41), and worry (indirect effect: b = -0.26, SE = 0.35, 95% Boot CI -0.68 – -0.04) — significantly mediated the effect of the intervention on insomnia severity at six-month follow-up. The indirect effect was greater through work-related rumination than through worrying, and the contrast between the size of the indirect effects was significant (estimate: 0.72, SE = 0.38, 95% Boot CI 0.07 – 1.57). The direct effect of the intervention to reduce insomnia remained significant after incorporating the two mediators in the model: (direct effect: b = -2.39, SE = 0.58, 95% CI -3.53 – -1.25). This indicates that the intervention also reduced insomnia, independent of its indirect effects on worry and work-related rumination."

18-i) Subgroup analysis of comparing only users

A subgroup analysis of comparing only users is not uncommon in ehealth trials, but if done, it must be stressed that this is a self-selected sample and no longer an unbiased sample from a randomized trial (see 16-iii).

	1	2	3	4	5	
subitem not at all important	○	○	○	○	○	essential

Does your paper address subitem 18-i?

Copy and paste relevant sections from the manuscript (include quotes in quotation marks "like this" to indicate direct quotes from your manuscript), or elaborate on this item by providing additional information not in the ms, or briefly explain why the item is not applicable/relevant for your study

Meine Antwort

19) All important harms or unintended effects in each group

(for specific guidance see CONSORT for harms)

Does your paper address CONSORT subitem 19? *

Copy and paste relevant sections from the manuscript (include quotes in quotation marks "like this" to indicate direct quotes from your manuscript), or elaborate on this item by providing additional information not in the ms, or briefly explain why the item is not applicable/relevant for your study

"At T2, no iCBT-I group member versus one control subject experienced reliable deterioration (T2 score of > 5.01 above the T1 score). At six-month follow-up, corresponding numbers were one and two."

19-i) Include privacy breaches, technical problems

Include privacy breaches, technical problems. This does not only include physical “harm” to participants, but also incidents such as perceived or real privacy breaches [1], technical problems, and other unexpected/unintended incidents. “Unintended effects” also includes unintended positive effects [2].

	1	2	3	4	5	
subitem not at all important	○	○	○	○	○	essential

Does your paper address subitem 19-i?

Copy and paste relevant sections from the manuscript (include quotes in quotation marks "like this" to indicate direct quotes from your manuscript), or elaborate on this item by providing additional information not in the ms, or briefly explain why the item is not applicable/relevant for your study

No privacy breaches and technical problems occurred.

19-ii) Include qualitative feedback from participants or observations from staff/researchers

Include qualitative feedback from participants or observations from staff/researchers, if available, on strengths and shortcomings of the application, especially if they point to unintended/unexpected effects or uses. This includes (if available) reasons for why people did or did not use the application as intended by the developers.

	1	2	3	4	5	
subitem not at all important	○	○	○	○	○	essential

Does your paper address subitem 19-ii?

Copy and paste relevant sections from the manuscript (include quotes in quotation marks "like this" to indicate direct quotes from your manuscript), or elaborate on this item by providing additional information not in the ms, or briefly explain why the item is not applicable/relevant for your study

"Only three participants reported their reason for dropout. Stated reasons were “technical problems”, “lack of time” and “already sufficient help before the last module”. "

DISCUSSION

22) Interpretation consistent with results, balancing benefits and harms, and considering other relevant evidence

NPT: In addition, take into account the choice of the comparator, lack of or partial blinding, and unequal expertise of care providers or centers in each group

22-i) Restate study questions and summarize the answers suggested by the data, starting with primary outcomes and process outcomes (use)

Restate study questions and summarize the answers suggested by the data, starting with primary outcomes and process outcomes (use).

	1	2	3	4	5	
subitem not at all important	○	○	○	○	○	essential

Does your paper address subitem 22-i? *

Copy and paste relevant sections from the manuscript (include quotes in quotation marks "like this" to indicate direct quotes from your manuscript), or elaborate on this item by providing additional information not in the ms, or briefly explain why the item is not applicable/relevant for your study

"The majority of studies on iCBT-I were not designed to reach the general working population, since they either targeted clinical samples or were conducted in an indicated prevention setting. Thus, there is insufficient evidence to confidently claim that iCBT-I is an effective tool for universal prevention among the general working population. For this purpose, we examined the efficacy of a self-help web-based recovery training among the general working population in the context of universal prevention.
The intervention's moderate-to-large effects on insomnia severity are consistent with results from recent meta-analyses that mainly focused on iCBT-I in clinical samples or in indicated prevention setting [27–30]. Effects were substantially larger than the expected effects of an iCBT-I intervention for universal prevention [37]. However, consistent with expectations, effects also were less than in previous studies evaluating the efficacy of this training when used as either a guided or self-guided version by a sample of teachers with elevated insomnia symptoms [34,35]."

22-ii) Highlight unanswered new questions, suggest future research

Highlight unanswered new questions, suggest future research.

	1	2	3	4	5	
subitem not at all important	○	○	○	○	○	essential

Does your paper address subitem 22-ii?

Copy and paste relevant sections from the manuscript (include quotes in quotation marks "like this" to indicate direct quotes from your manuscript), or elaborate on this item by providing additional information not in the ms, or briefly explain why the item is not applicable/relevant for your study

"Finally, no measures were assessed to determine which of the training elements were the most effective and which elements less so. However, results from mediation analysis suggest that techniques that reduce pre-sleep cognitive activation played an important role in the training´s efficacy. To explore which of the intervention's components work best, further research is needed."

20) Trial limitations, addressing sources of potential bias, imprecision, and, if relevant, multiplicity of analyses

20-i) Typical limitations in ehealth trials

Typical limitations in ehealth trials: Participants in ehealth trials are rarely blinded. Ehealth trials often look at a multiplicity of outcomes, increasing risk for a Type I error. Discuss biases due to non-use of the intervention/usability issues, biases through informed consent procedures, unexpected events.

	1	2	3	4	5	
subitem not at all important	○	○	○	○	○	essential

Does your paper address subitem 20-i? *

Copy and paste relevant sections from the manuscript (include quotes in quotation marks "like this" to indicate direct quotes from your manuscript), or elaborate on this item by providing additional information not in the ms, or briefly explain why the item is not applicable/relevant for your study

"The following limitations of this trial must be acknowledged. First, due to the restricted funding period, the final sample size differed from the sample size initially intended. However, given the intervention's sizeable effect on the primary outcome and the findings of sensitivity analysis, we believe that our conclusions are not substantially affected by the smaller sample size. Second, the effects were observed within the confines of a randomized controlled trial, a study architecture typically associated with a highly-structured study protocol (e.g., a strict screening process), which is unlikely to be found in routine occupational health care. Therefore, it is unclear if our results can be generalized to routine occupational health care settings. This said, the present results are a first indication of the efficacy of iCBT-I in a universal-prevention setting, where inclusion and exclusion criteria are minimized and every worker motivated to undertake the training can participate. Finally, no measures were assessed to determine which of the training elements were the most effective and which elements less so. However, results from mediation analysis suggest that techniques that reduce pre-sleep cognitive activation played an important role in the training´s efficacy. To explore which of the intervention's components work best, further research is needed."

21) Generalisability (external validity, applicability) of the trial findings

NPT: External validity of the trial findings according to the intervention, comparators, patients, and care providers or centers involved in the trial

21-i) Generalizability to other populations

Generalizability to other populations: In particular, discuss generalizability to a general Internet population, outside of a RCT setting, and general patient population, including applicability of the study results for other organizations

	1	2	3	4	5	
subitem not at all important	○	○	○	○	○	essential

Does your paper address subitem 21-i?

Copy and paste relevant sections from the manuscript (include quotes in quotation marks "like this" to indicate direct quotes from your manuscript), or elaborate on this item by providing additional information not in the ms, or briefly explain why the item is not applicable/relevant for your study

"Moreover, the GET.ON Recovery training's efficacy was not restricted to those with more severe insomnia symptoms at baseline. In this way, the present findings strengthen arguments as to the generalizability and robustness of prior results on this training´s efficacy [34,35]."

21-ii) Discuss if there were elements in the RCT that would be different in a routine application setting

Discuss if there were elements in the RCT that would be different in a routine application setting (e.g., prompts/reminders, more human involvement, training sessions or other co-interventions) and what impact the omission of these elements could have on use, adoption, or outcomes if the intervention is applied outside of a RCT setting.

	1	2	3	4	5	
subitem not at all important	○	○	○	○	○	essential

Does your paper address subitem 21-ii?

Copy and paste relevant sections from the manuscript (include quotes in quotation marks "like this" to indicate direct quotes from your manuscript), or elaborate on this item by providing additional information not in the ms, or briefly explain why the item is not applicable/relevant for your study

"Second,the effects were observed within the confines of a randomized controlled trial, a study architecture typically associated with a highly-structured study protocol (e.g., a strict screening process), which is unlikely to be found in routine occupational health care. Therefore, it is unclear if our results can be generalized to routine occupational health care settings. This said, the present results are a first indication of the efficacy of iCBT-I in a universal-prevention setting, where inclusion and exclusion criteria are minimized and every worker motivated to undertake the training can participate."

OTHER INFORMATION

23) Registration number and name of trial registry

Does your paper address CONSORT subitem 23? *

Copy and paste relevant sections from the manuscript (include quotes in quotation marks "like this" to indicate direct quotes from your manuscript), or elaborate on this item by providing additional information not in the ms, or briefly explain why the item is not applicable/relevant for your study

German Register Clinical Trials: DRKS00006223
https://www.drks.de/drks_web/

24) Where the full trial protocol can be accessed, if available

Does your paper address CONSORT subitem 24? *

Cite a Multimedia Appendix, other reference, or copy and paste relevant sections from the manuscript (include quotes in quotation marks "like this" to indicate direct quotes from your manuscript), or elaborate on this item by providing additional information not in the ms, or briefly explain why the item is not applicable/relevant for your study

Not available

25) Sources of funding and other support (such as supply of drugs), role of funders

Does your paper address CONSORT subitem 25? *

Copy and paste relevant sections from the manuscript (include quotes in quotation marks "like this" to indicate direct quotes from your manuscript), or elaborate on this item by providing additional information not in the ms, or briefly explain why the item is not applicable/relevant for your study

The European Union funded this study, project number: EFRE: CCI 2007DE161PR001.

X27) Conflicts of Interest (not a CONSORT item)

X27-i) State the relation of the study team towards the system being evaluated

In addition to the usual declaration of interests (financial or otherwise), also state the relation of the study team towards the system being evaluated, i.e., state if the authors/evaluators are distinct from or identical with the developers/sponsors of the intervention.

	1	2	3	4	5	
subitem not at all important	○	○	○	○	○	essential

Does your paper address subitem X27-i?

Copy and paste relevant sections from the manuscript (include quotes in quotation marks "like this" to indicate direct quotes from your manuscript), or elaborate on this item by providing additional information not in the ms, or briefly explain why the item is not applicable/relevant for your study

Dirk Lehr and Daniel David Ebert are stakeholders in the ‘Institute for Online Health Training’, which aims to transfer scientific knowledge related to the present research into routine health care. Dörte Behrendt and Kai Spiegelhalder do not indicate any conflicts of interest.

About the CONSORT EHEALTH checklist

As a result of using this checklist, did you make changes in your manuscript? *

- ◯ yes, major changes
- ◉ yes, minor changes
- ◯ no

What were the most important changes you made as a result of using this checklist?

Meine Antwort

How much time did you spend on going through the checklist INCLUDING making changes in your manuscript *

Meine Antwort

As a result of using this checklist, do you think your manuscript has improved? *

- ◉ yes
- ◯ no
- ◯ Sonstiges:

Would you like to become involved in the CONSORT EHEALTH group?

This would involve for example becoming involved in participating in a workshop and writing an "Explanation and Elaboration" document

- ○ yes
- ◉ no
- ○ Sonstiges:

Any other comments or questions on CONSORT EHEALTH

Meine Antwort

STOP - Save this form as PDF before you click submit

To generate a record that you filled in this form, we recommend to generate a PDF of this page (on a Mac, simply select "print" and then select "print as PDF") before you submit it.

When you submit your (revised) paper to JMIR, please upload the PDF as supplementary file.

Don't worry if some text in the textboxes is cut off, as we still have the complete information in our database. Thank you!

Final step: Click submit !

Click submit so we have your answers in our database!

SENDEN

Geben Sie niemals Passwörter über Google Formulare weiter.

Dieser Inhalt wurde nicht von Google erstellt und wird von Google auch nicht unterstützt. Missbrauch melden - Nutzungsbedingungen

Google Formulare
